# Genetically predicted circulating levels of cytokines and the risk of depression: a bidirectional Mendelian-randomization study

**DOI:** 10.3389/fgene.2023.1242614

**Published:** 2023-08-04

**Authors:** Meiti Wang, Guixiang Jin, Ying Cheng, Shi-Yang Guan, Jinxin Zheng, Shun-Xian Zhang

**Affiliations:** ^1^ Shanghai Mental Health Center, Shanghai Jiao Tong University School of Medicine, Shanghai, China; ^2^ Shanghai Yangpu Mental Health Center, Shanghai, China; ^3^ Second Affiliated Hospital of Anhui Medical University, Hefei, Anhui, China; ^4^ School of Global Health, Chinese Center for Tropical Diseases Research—Shanghai Jiao Tong University School of Medicine, Shanghai, China; ^5^ Clinical Research Center, Longhua Hospital, Shanghai University of Traditional Chinese Medicine, Shanghai, China

**Keywords:** major depressive disorder, inflammatory cytokine, Mendelian randomization, GWAS, IL-18, RANTES, IL-1beta

## Abstract

**Objective:** Inflammatory cytokines disturbance is the main result of immune dysregulation, which is widely described in major depressive disorder (MDD). However, the potential causal relationship between these two factors has not been discovered. Therefore, the purpose of this study was to investigate the causal relationship between inflammatory cytokines and MDD risk by using the two-sample Mendelian randomization (MR) analysis.

**Method:** Two genetic instruments obtained from publicly available gene profile data were utilized for the analysis. We obtained the genetic variation data of 41 inflammatory cytokines from genome-wide association studies (GWAS) meta-analysis of 8293 individuals of Finnish descent. The MDD data, including 135,458 MDD cases and 344,901 controls, were obtained from the Psychiatric Genomics Consortium Database. For the Mendelian randomization (MR) estimation, several methods were employed, namely, MR-Egger regression, inverse-variance weighted (IVW), weighted median, and MR-Pleiotropy RESidual Sum and Outlier (MR-PRESSO) methods.

**Result:** A causal relationship was identified between the genetically proxied levels of Interleukin (IL) −18, IL-1β, and Regulated upon activation normal T cell expressed and secreted (RANTES) and the risk of MDD (OR = 0.968, 95%CI = 0.938, 0.998, *p* = 0.036; OR = 0.875, 95%CI = 0.787, 0.971, *p* = 0.012; OR = 0.947, 95%CI = 0.902, 0.995, *p* = 0.03; respectively). However, our Mendelian randomization (MR) estimates provided no causality of MDD on inflammatory cytokines.

**Conclusion:** Our study elucidates the connection between inflammatory cytokines and MDD by using MR analysis, thereby enhancing our comprehension of the potential mechanisms. By identifying these associations, our findings hold substantial implications for the development of more effective treatments aimed at improving patient outcomes. However, further investigation is required to fully comprehend the exact biological mechanisms involved.

## 1 Introduction

Major depressive disorder (MDD) is a prevalent and debilitating mental illness illustrated by hopelessness, persistent feelings of sadness, and loss of interest or pleasure. The global prevalence rate of depression is about 4.4%, with higher rates reported in women and those with comorbid medical conditions (World Health Organization, 2017). The number of disability adjusted life years (DALYs) due to depression has steadily increased in certain age groups, which has hindered social and economic development and placed a significant burden on the public health sector (GBD, 2019 Diseases and Injuries Collaborators, 2020). Multiple biological features that have been implicated in depression include imbalances in neurotransmitters such as serotonin, dopamine, and norepinephrine, as well as dysfunction of the hypothalamic-pituitary-adrenal (HPA) axis (Krishnan and Nestler, 2008). Additionally, inflammatory dysregulation have been progressively identified as important contributors to depression, as well as genetic and epigenetic factors (Miller and Raison, 2016). Although the past decade has carried remarkable scientific progress, the underlying mechanisms and etiology of depression remain unclear, making the development of effective preventive and therapeutic interventions and prognosis assessments challenging.

Numerous evidence suggests that proinflammatory cytokines, such as interleukin (IL)-6, IL-1β, and tumor necrosis factor alpha (TNF-α), are elevated in individuals with depressive disorder (Haapakoski et al., 2015). On the contrary, anti-inflammatory cytokines, including IL-4, IL-13, and IL-10, are found to be decreased in individuals with depression. These findings support the concept of an imbalance in the cytokine network, favoring a pro-inflammatory state in individuals with depression (Köhler et al., 2014). Additionally, antidepressant treatment has also been found to modulate the immune response by reducing the protein concentrations of inflammatory cytokines and increasing the protein concentrations of anti-inflammatory cytokines (Rosenblat et al., 2014). However, the levels of inflammatory cytokines in depression are complicated and inconsistent across studies. The levels of IFN-γ have yielded conflicting results in depression compared to healthy subjects, with a meta-analysis study finding a lower difference and a study identifying significantly higher concentrations in patients with MDD (Köhler et al., 2017; Lee et al., 2020). Some studies discovered that higher levels of IL-6 and IL-1β are correlated with more severe depressive symptoms, while others have found no significant association (Dowlati et al., 2010; Kagawa et al., 2017; Köhler et al., 2017). Inconsistencies in study findings may cause by differences in the study design, patient population, and measurement techniques. Moreover, observational studies that establish a link between specific circulating inflammatory cytokine concentrations and the risk of depression often suffer from limited sample sizes, restricting the generalizability of their findings. Hence, it is imperative to examine the direct connection between immune inflammation and depression and to verify methods for confounding factor mitigation.

Mendelian randomization (MR) analysis is a statistic technique, which employs single nucleotide polymorphisms (SNPs) as instrumental variables (IVs) to evaluate the causative impact of modifiable risk factors on population health, and not subject to the same biases as environmental exposures or lifestyle factors. This method utilizes the random assortment of genes during meiosis, which is akin to a natural experiment and not influenced by confounding and reverse causation, causing MR analyses less sensitive to reverse causation in clinical studies due to the fact that genotypes precede the onset of diseases (Davey Smith and Hemani, 2014). Consequently, it has become widely used to estimate the potential causal associations between exposures and diseases. In this study, MR was employed to examine the genetically levels of cytokine over the lifespan, with special emphasis on capturing variations attributed to genetic factors, rather than epigenetic changes that affect gene expression. In this present study, we utilized publicly available GWAS data and employed a two-sample MR analysis to elucidate the potential bidirectional relationship between inflammatory cytokines and MDD.

## 2 Methods

### 2.1 Study design

**FIGURE 1:**
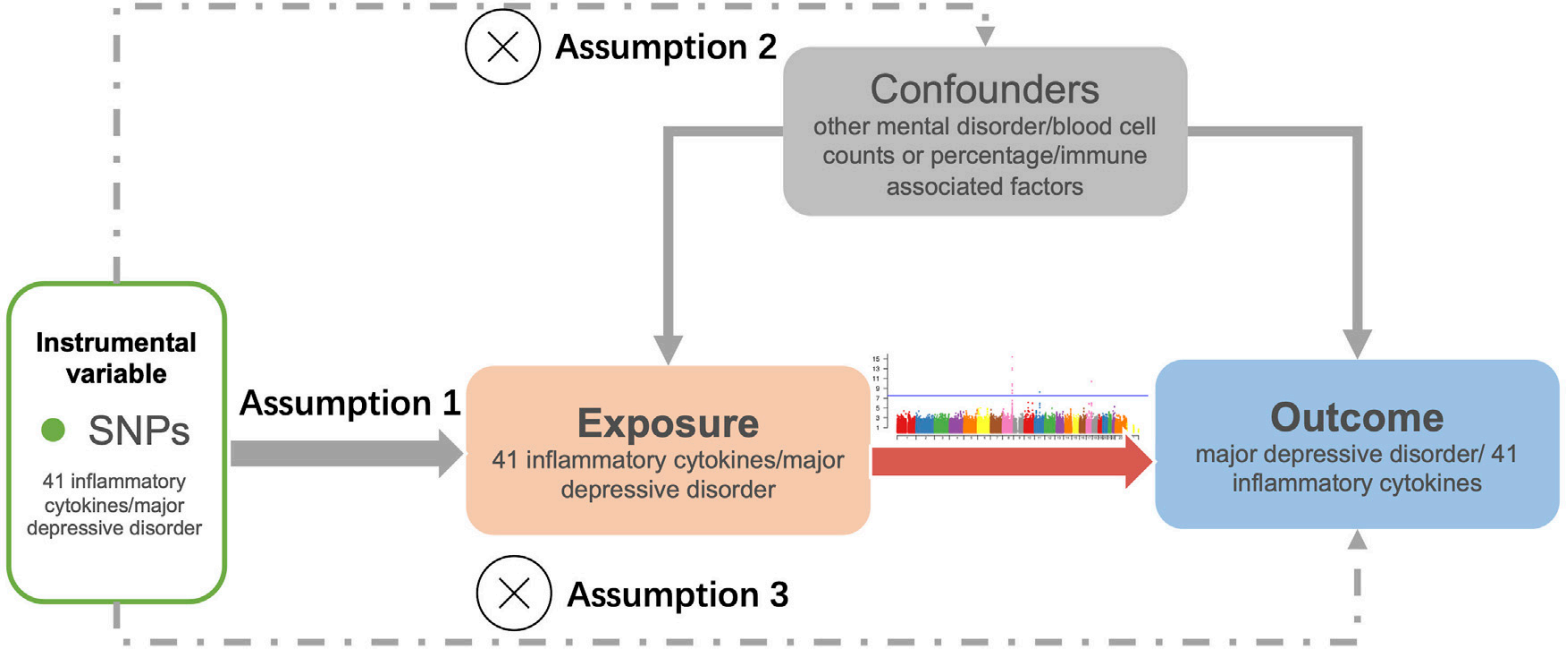
Flowchart of the study. The three assumptions of the MR study. Assumption 1: The genetic variation used as an instrumental variable is associated with the exposure. Assumption 2: The genetic variation is independent of confounding factors. Assumption 3: The genetic variation affects the outcome solely through the exposure and does not operate through other pathways.

The study design for bidirectional MR analysis is illustrated in Figure 1. MR analysis rests on three crucial assumptions: 1) the genetic variation selected as the IV is genuinely correlated with the exposure of interest; 2) the genetic variation must not be related with any confounding factors; 3) the genetic variation only affects outcome through exposure (Lawlor et al., 2008).

In this study, we utilized two sets of Genome-wide association studies (GWAS) databases concerning about 41 systemic inflammatory factors and MDD. Firstly, we infer the causal relationship between inflammatory factors and depression by using the genetic variation related to each inflammatory factor. Secondly, we explored the genetic variation related to depression, to infer the causal relationship between depression and inflammatory factors. All the GWAS data used in this study were derived from European ancestry. And this study constitutes a second analysis of previously published data. Therefore, no additional ethics approval was necessary.

### 2.2 GWAS summary data for cytokines

The summary-level data and IVs adjusted for age, sex, and body mass index for 41 peripheral inflammatory cytokines were derived from the most comprehensive GWAS data including 8293 Finnish individuals from the Cardiovascular Risk in Young Finns Study (YFS) and the FINRISK studies (FINRISK 2002 and FINRISK 1997) (Ahola-Olli et al., 2017). The survey was conducted in Finland, with participants randomly selected from five different geographical areas and ranging in age from 25 to 74. The cytokines were quantified using Bio-Rad’s premixed Bio-Plex Pro Human Cytokine 27-plex Assay and 21-plex Assay, and a Bio-Plex 200 reader with Bio-Plex 6.0 software. Specifically, EDTA plasma was used for quantification in FINRISK 1997, heparin plasma in FINRISK 2002, and serum in YFS. For the analysis, only the measured values of each cytokine within the detectable range are considered. In addition, cytokines with deletion values over 90%, especially 7 out of the 48 cytokines, were excluded from the study. The written informed consent was documented in the original article. The GWAS Catalog server at https://www.ebi.ac.uk/gwas/home provided the full GWAS summary statistics.

To investigate the causal relationship between systemic inflammation and depression, we chosen SNPs that strongly predicted the importance of cytokines at genome-wide significance levels (linkage disequilibrium (LD] *r* ^2^ < 0.01, *p* < 5 × 10^−8^). Since only a limited number of cytokines had more than two independent SNPs at this level of significance, we adopted an alternative threshold of *p* < 5 × 10^−6^ to include more SNPs as IVs (Supplementary Table S1), which has been previously suggested (Burgess et al., 2013). SNPs related to potential confounding factors reported in previous research, which are correlated with depressive disorder, such as other mental disorders (schizophrenia, bipolar disorder, personality disorder, et al.) (Cross-Disorder Group of the Psychiatric Genomics Consortium. Electronic address: plee0@mgh.harvard.edu and Cross-Disorder Group of the Psychiatric Genomics Consortium, 2019), antidepressant treatment (Chauquet et al., 2021), age (Labrecque and Swanson, 2019), and gender (Kamitaki et al., 2020), should be excluded from the analysis. To estimate whether the selected SNP were related to the potential confounding factors, we utilized Phenoscanner (www.Phenoscanner.medschl.cam.ac.uk), which shows the comprehensive data of the association between genotype and phenotype (Supplementary Table S2). Additionally, we deleted several SNPs related to various cytokines to avoid pleiotropy (Supplementary Table S3). To estimate the proportion of variance in exposure explained by each SNP and to avoid weak instrument bias, we calculated the *R* ^2^ value and F-statistic respectively (Palmer et al., 2012). Typically, an F-statistic greater than 10 is considered effective in attenuating bias caused by weak IVs.

### 2.3 GWAS summary data for depressive disorder

The GWAS data from a meta-analysis, including 135,458 cases of MDD and 344,901 controls, were adjusted for age and sex, and the meta-analysis reported significant correlation of 44 SNPs with MDD [*p* < 5 × 10^−8^, linkage disequilibrium (LD) r2 < 0.01] (Wray et al., 2018). Collectively, these SNPs accounted for 0.23% of the variability in MDD. The confounding factors considered in this part included blood cell counts or percentages, immune-associated factors, and immune-related diseases that have been associated with inflammatory cytokines (Medzhitov, 2008; Zhernakova et al., 2009). By using the PhenoScanner platform, four cytokines were deleted from the study because of correlating with the confounders, then only 39 SNPs associated with MDD were used in this study (Supplementary Table S4). And after excluding the palindromic sequence SNPs, a total of 30 SNPs were selected for further analysis (Supplementary Table S5). The F-statistic, which was used to evaluate the predictive power of the instruments, surpassing the conventional threshold of 10, indicating a strong potential for predicting MDD (Supplementary Table S4). The full GWAS summary statistics were obtained from the Psychiatric Genomics Consortium (PGC) server at https://pgc.unc.edu/for-researchers/download-results/.

### 2.4 Statistical analysis

Following the harmonization of effect alleles across the GWASs of inflammatory cytokines and MDD, 3 MR analyses were conducted using different assumptions to calculate MR estimates. The analyses included the inverse-variance weighted (IVW), MR Egger, and weighted median methods. IVW was selected as the primary outcome among the three analyses due to its high efficiency and statistical power. However, it is based on the assumption that all genetic variants are valid IVs, which cannot always be true (Bowden et al., 2015). The weighted median approach serves as a compensation for the limitations of IVW, as it only necessitates that at least half of the weights align with valid IVs to generate credible estimates, instead of requiring all of them. On the other hand, the MR-Egger approach, as a test method to measure directional polymorphism (significance threshold: *p* < 0.05), requires that pleiotropic effect must be independent of the relationship between variant and exposure (Ong and MacGregor, 2019).

Due to the heterogeneity of MR estimation will be greatly compromised, sensitivity analysis plays a crucial role in MR research to determine the potential pleiotropy and heterogeneity. Firstly, IVW and MR-Egger regression were used, and Cochran Q statistics (Cochran Q-derived *p* < 0.05) were generated to quantify heterogeneity. Secondly, The horizontal pleiotropy was determined by the MR-Egger intercept method, which calculates the intercept term after linear regression analysis. We employed MR-Pleiotropy RESidual Sum and Outlier methods (MR-PRESSO) to evaluate and address potential horizontal pleiotropy. MR-PRESSO comprises three parts: 1) horizontal pleiotropy detection, 2) horizontal pleiotropy correction through outlier removal, and 3) testing the significant differences of causality estimation before and after outlier correction. MR-PRESSO exhibits reduced bias and improved precision compared to IVW and MR-Egger when the percentage of horizontal pleiotropy variants is below 10%. We also conducted a leave-one-out analysis involving 25 iterations to assess whether the MR estimate was influenced or biased by individual SNPs.

## 3 Results

### 3.1 Genetic prediction of circulating cytokines for depression risk

Under the less stringent threshold of *p* < 5 × 10^−6^, all 41 cytokines exhibited independent single nucleotide polymorphisms (SNPs), with the identification and subsequent deletion of 17 duplicated SNPs (Supplementary Table S3). At the genome-wide significance level, 2 to 22 independent SNPs were identified as IVs for cytokines, with corresponding F-statistics ranging from 19.28 to 124.21 (Supplementary Table S1). In the phenoscanner research, eight SNPs in cytokines (rs11551183, rs117509142, rs2673604, rs396960, rs4737732, rs5754733, rs7088799, and rs9267091) that showed correlations with confounders or outcomes were excluded from the study.

**FIGURE 2:**
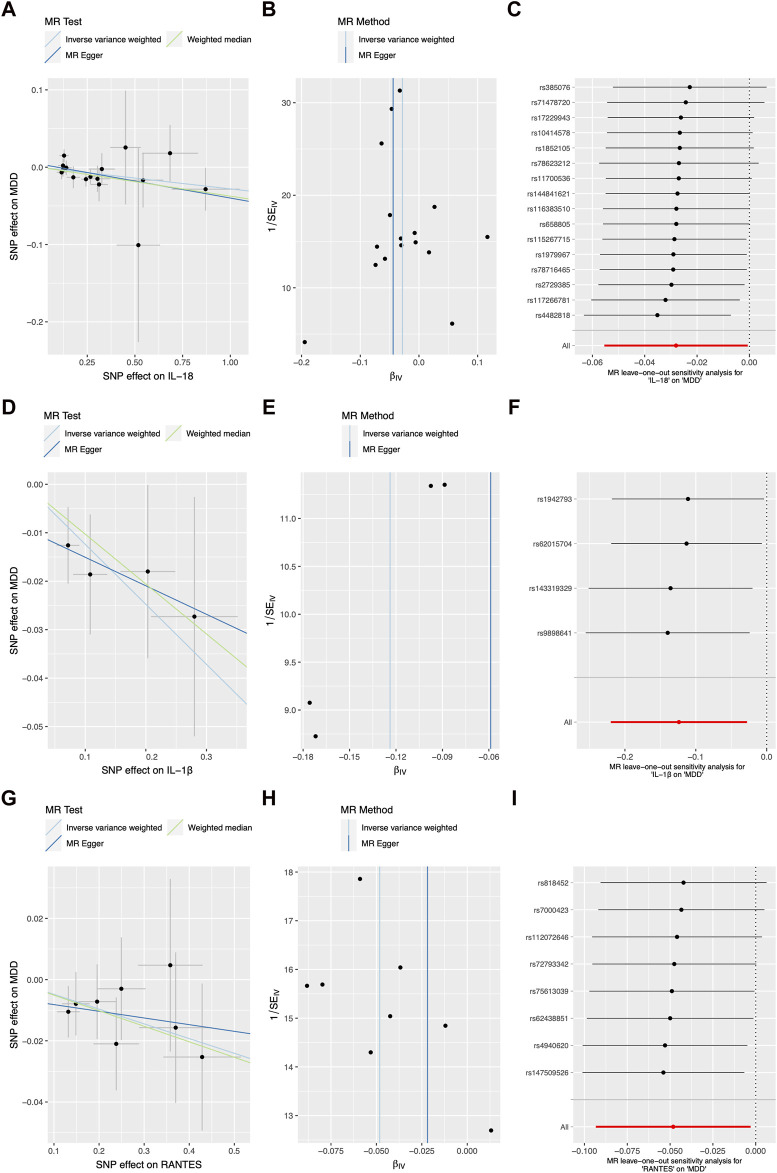
The scatter plots, funnel plots, and forest plots represented the SNPs of IL-18 **(A–C)**, IL-1β **(D–F)**, and RANTES **(G–I)** in relation to inflammatory cytokines and the associated risk of major depressive disorder, with outliers removed using MR-PRESSO. Abbreviation: MR Mendelian randomization, SNP single-nucleotide polymorphism, IL interleukin, RANTES reduced upon activation normal T cell expressed and secreted.

Figure 2 and Table 1 illustrates the correlation between genetically predicted systemic inflammatory cytokines and MDD. Using the IVW method, genetically proxied concentrations of IL-18, IL-1β, and reduced upon activation normal T cell expressed and secreted (RANTES) were found to be inversely associated with a increased risk of depression (OR = 0.968, 95%CI = 0.938, 0.998, *p* = 0.036; OR = 0.875, 95%CI = 0.787, 0.971, *p* = 0.012; OR = 0.947, 95%CI = 0.902, 0.995, *p* = 0.03; respectively). The association of IL-18 showed no statistically significant effect in the MR-Egger regression (OR = 0.948, 95%CI = 0.892, 1.0101, *p* = 0.11), but it exhibited a modest statistically significant in weighted median approaches (OR = 0.959, 95%CI = 0.918, 0.999, *p* = 0.049). And The association of IL-1β, and RANTES with the outcome was not statistically significant in the MR-Egger regression (OR = 0.914, 95%CI = 0.737, 1.132, *p* = 0.502; OR = 0.989, 95%CI = 0.872, 1.12, *p* = 0.869; respectively) and weighted median approaches (OR = 0.898, 95%CI = 0.779, 1.031, *p* = 0.134; OR = 0.954, 95%CI = 0.894, 1.02, *p* = 0.152; respectively). The beta coefficients obtained through the MR-Egger method and the weighted median method consistently align with the beta coefficients from the IVW analysis (see Table 1), thus supporting the meaningfulness of the results. The MR estimate results of the other inflammatory cytokines were shown in Supplementary Figure S1.

**TABLE 1:** Estimation of associations between circulating IL-18, IL-1β, and RANTES and risk of MDD using Mendelian randomization analysis.

Methods	MR results				Heterogeneity test			Horizontal pleiotropy test			
					Cochrane Q test			MR-egger intercept test		MR-PRESSO global test	
	SNP	Beta	SE	*p*	Q	df	*p*	Intercept	*p*	RSSobs	*p*
IL-18											
IVW	15	−0.033	0.016	0.036	6.023	14	0.966			12.796	0.825
MR Egger	15	−0.053	0.031	0.109	5.456	13	0.963	0.005	0.465		
Weighted median	15	−0.042	0.021	0.05							
IL-1β											
IVW	4	−0.134	0.053	0.012	1.361	3	0.715			2.178	0.797
MR Egger	4	−0.089	0.11	0.502	1.15	2	0.563	−0.006	0.691		
Weighted median	4	−0.108	0.072	0.134							
RANTES											
IVW	9	−0.054	0.025	0.03	4.634	8	0.796			8.413	0.65
MR Egger	9	−0.011	0.064	0.869	4.119	7	0.766	−0.01	0.496		
Weighted median	9	−0.047	0.033	0.152							

IVW, MR, egger, and Weighted median were utilized for the MR, analysis. Heterogeneity between SNP-specific estimates was assessed using the Cochran Q-test, and evidence of polymorphism was tested using MR-Egger regression and MR-PRESSO., Abbreviation; MR, mendelian randomization; IVW, inverse variance weighted; SNP, single-nucleotide polymorphism; SE, standard error, df degree of freedom; MR-PRESSO, Mendelian randomization Pleiotropy RESidual Sum and Outlier, RSSobs, observed residual sum of squares; IL, interleukin; RANTES, reduced upon activation normal T cell expressed and secreted.

In the sensitivity analysis shown in Figure 2 and Table 1, both the Cochran Q-test based on MR-Egger and IVW showed no significant heterogeneity for IL-18, IL-1β, and RANTES (all *p* values >0.05). Additionally, there was no evidence of a significant intercept shown in Table 1 (intercept = 0.005, SE = 0.006, *p* = 0.465; intercept = −0.006, SE = 0.014, *p* = 0.69; intercept = −0.01, SE = 0.014, *p* = 0.496; respectively), indicating the absence of directional pleiotropy. No individual SNP with a strong influence on the overall effect was detected in the Leave-one-out sensitivity analyses (Figure 1). The sensitivity analysis results of the other inflammatory cytokines were shown in Supplementary Figure S1.

### 3.2 Genetic prediction of depression for risk of circulating cytokines

A total of 39 independent genome-wide significant SNPs were utilized as IVs for the study of depression. In the phenoscanner research, five SNPs (rs115507122, rs11643192, rs159963, rs5758265, and rs9427672) associated with depression were excluded from the study due to their correlations with either the confounders or the outcomes.

The IVW method revealed a significant upregulation in the risk for MCP-3 and TNFβ associated with MDD (OR = 0.567, 95% CI = 0.343, 0.938, *p* = 0.027; OR = 0.571, 95% CI = 0.376, 0.867, *p* = 0.009). However, contrasting results were obtained when employing the MR-Egger approach (OR:1.543, 95% CI = 0.263, 9.061, *p* = 0.635; OR:1.243, 95% CI = 0.287, 5.372, *p* = 0.773) and weighted median approaches (OR: 0.577, 95% CI = 0.293, 1.134, *p* = 0.111; OR: 0.589, 95% CI = 0.318, 1.086, *p* = 0.09). The inconsistent beta direction of these 3 MR methods indicates that there was no genetic causal relationship from MDD to MCP-3 and TNFβ. The result of the individual causal estimates and MR regression slopes were depicted in Supplementary Figure S2.

## 4 Discussions

The relationship between depression and cytokines has remained unclear, with conflicting findings. By implementing this MR analysis, we were able to surpass limitations and establish a study design, that is, more robust, enabling a confident investigation of causality. This is the first comprehensive bidirectional MR study to investigate the association between genetically determined inflammatory cytokines and depression. By analyzing 41 cytokines using the largest available GWAS datasets, we identified that a potential causal relationship between IL-18, IL-1β, and RANTES and depression. However, we did not find sufficient evidence to support a causal relationship between genetically predicted depression and the risk of inflammatory cytokines.

Since depression is commonly associated with low levels of inflammation and there appears to be a relatively high prevalence of elevated blood levels of cytokines among patients with depression. The MR result appears to contradict the biological processes related to depression and immune function and partially consistent with previous studies and partially contradictory. Firstly, our study revealed a significant inverse association between genetically proxied IL-1β levels and MDD. IL-1β is a pro-inflammatory cytokine induced by immune cells in response to microbial molecules, and it promotes inflammation by initiating and amplifying inflammatory responses (Lopez-Castejon and Brough, 2011). Studies have confirmed that IL-1β can mediate glutamate overproduction and reduce serotonin reuptake by modulating mitochondria glutaminase activity, which contributes to the progress of depression (van den Biggelaar et al., 2007). Moreover, numerous clinical studies have reported elevated levels of IL-1β in patients with MDD (Zou et al., 2018; Jin et al., 2020). However, contrary to the results of some experiments and clinical studies, our study demonstrated that there is an inverse relationship between the genetically proxied circulating IL-1β levels and MDD. One possible reason for this difference is that IL-1β has pleiotropic effects due to its cellular localization and different stages of depression. This is supported by a clinical study that examined MDD patients with a chronic course of illness, which found no difference in the protein concentration of IL-1β (Cassano et al., 2017). And in a meta-analysis, IL-1β cannot represent the state of mood episodes (Solmi et al., 2021). Moreover, a study by Kobayashi found that neither the blood mRNA levels nor the protein levels of IL-1β exhibited elevation in the MDD group (Kobayashi et al., 2022).

Similarly, our study also revealed a significant inverse association between genetically proxied IL-18 concentrations and major depressive disorder (MDD). IL-18 is a proinflammatory cytokine which belongs to the IL-1 family is involved in the activation of different types of immune cells, playing a role in immune responses and inflammation (Kaplanski, 2018). The upregulated activity of the Nod-like receptor pyrin containing protein 3 (NLRP3) inflammasome promotes the maturation and release of IL-18, leading to neuroinflammation, which can contribute to depressive symptoms (Kouba et al., 2022). A comprehensive meta-analysis reported a significant increase in IL-18 concentrations among individuals with depression (Köhler et al., 2017). Although our MR results are opposite to previous findings, they are still consistent with certain hypotheses and published studies. For instance, a recent study revealed a negative association between IL-18 concentrations and the total HAMD-17 score, specifically in female patients with MDD (Min et al., 2023). Additionally, IL-18-deficient mice exhibited significant destructions in learning and memory function, as well as reduced motivation, resulting in depressive-like behaviors (Yamanishi et al., 2021).

In addition, an inverse association was also found between genetically proxied RANTES and MDD. RANTES, a chemokine protein, plays a crucial role in immune responses and inflammation processes and attracting immune cells to sites of inflammation and infection (Appay and Rowland-Jones, 2001). The current evidence from clinical and experimental studies does not support such an inverse association. For instance, a separate study reported higher levels of RANTES in depressed patients when compared to control subjects (Małujło-Balcerska et al., 2022). Additionally, RANTES is a clock-controlled gene (CCG) involved in the clock-immunological mechanisms that contribute to the effects of Per2 on depression-like behavior induced by neuroinflammation (Chen et al., 2020). Furthermore, our study aligns with a limited study, which found that the RANTES level of female MDD patients with suicidal thoughts was lower than that in female patients without suicidal thoughts (Grassi-Oliveira et al., 2012). The reason for those discrepancies can be attributed to the limitations of previous cross-sectional or prospective observational studies, which were frequently constrained by small sample sizes and the heterogeneity of the recruited patients. Furthermore, MR primarily investigates genetic associations, while the conversion of genetic information to proteins undergoes intricate processes, including transcription, modification, and epigenetic modification (Need and Goldstein, 2009). As a result, disparities may arise between the findings at the genetic and protein levels. Additionally, there are multiple neurobiological pathways upstream or downstream that can influence cytokine levels (Becher et al., 2017). Those proteins interaction can lead to the introduction of biases. Furthermore, it is important to emphasize that antidepressant treatment can introduce additional variability in cytokine concentrations, which may contribute to observed differences (Liu et al., 2020). It is also necessary to point out that the relationship between the immune system and psychosis is intricate. For example, CRP has long been considered a risk factor for schizophrenia, exhibiting positive correlations with cognitive impairment, negative symptoms, and metabolic syndrome in SCZ patients (Jauhar et al., 2022). However, contrary to these beliefs, a large meta-analysis has revealed that CRP might have a protective effect against schizophrenia. Furthermore, a recent MR study has demonstrated that genetically proxied high levels of CRP significantly mitigate the risk of schizophrenia (Lin et al., 2019). Nevertheless, the precise underlying mechanism connecting CRP and schizophrenia remains unclear. Thus, the mechanism between genetically proxied inflammatory cytokines and depression, similar to that of CRP and schizophrenia, needs further studying.

This study found no evidence that the genetic prediction of MDD is related to the expression of systemic inflammatory regulators. Although the results of this study did not indicate a genetic correlation between depression and inflammatory cytokines, it was important to note that depression may still influence on the progression of inflammation which did not investigate within the extent of this study. For example, depression can straightly affect endocrine and immune processes, inhibit the activities of natural killer cells and DNA repair enzymes (Park et al., 2015; Miller and Raison, 2016). Several observational studies demonstrated that inflammatory cytokines were upregulated in first-episode drug naïve MDD patients and downregulated to normal levels after antidepressant treatment (Lee et al., 2020; Lan et al., 2021). However, it is important to note that these observational studies were predominantly cross-sectional or case-control in nature. Cross-sectional studies can only reveal associations without establishing causal relationships, and numerous factors can influence the production of cytokines, containing the complex cytokine networks rather than the disease itself.

Our study possesses a number of notable strengths. Firstly, we conducted analyses that encompassed a wide range of inflammatory cytokines, ensuring comprehensive coverage of the subject matter. Additionally, most of our analyses benefited from a large sample size, which further enhances the robustness of our findings. Secondly, the utilization of a genetic instrumental variable provides a significant strength to our study. This approach enabled us to conduct a comprehensive MR analysis, investigating the relationship between inflammatory factors and the risk of depression. This innovative endeavor holds considerable significance in understanding whether elevated or reduced inflammatory cytokines could instigate changes in the risk of developing depression, and whether the genetic predisposition to depression can change the changes of circulating inflammatory factors. Thirdly, by employing the MR design, this study effectively emulates the randomized controlled trials (RCT) within an observational background. RCT are widely accepted as the gold standard for studying causality, but they often entail high costs and practical challenges. However, MR studies offer a valuable avenue to alleviate confounding biases because of SNPs are randomly assigned at pregnancy. Lastly, the genetic instruments employed in this study were independent of the confounding factors in the relationship between exposure and results. Moreover, we also adjusted the genetic principal components to mitigate collider deviation that has the potential to violate fundamental MR assumptions.

It is important to highlight that our study had several limitations. Firstly, it is crucial to acknowledge that MR is specifically designed to estimate the long-term impact of a risk factor. The GWAS employed in the present analyses primarily focused on middle-aged individuals. It is plausible to hypothesize that the genetic mechanisms underlying the variation in cytokine blood levels during childhood differ from those governing cytokine blood concentration in older individuals (Labrecque and Swanson, 2019). Secondly, utilizing a more stringent cut-off value of *p* < 5 × 10^−8^ resulted in only 10 SNPs that exhibited genome-wide significance, we employed a significance cut-off of *p* < 5 × 10^−6^. Thirdly, there is no statistical significance in the estimation of MR-Egger and Weight Median in this study. Ideally, significant results across all three methods would be preferable. However, compared with other MR methods, especially MR-Egger, the statistical ability of IVW methods is significantly higher (Lin et al., 2021). It is necessary to emphasize the importance of consistent beta directionality in MR analyses, which we adhered to in this study (Bowden et al., 2016; Chen et al., 2022). Therefore, the results can still be considered as meaningful under the condition that the beta direction is consistent, although no significant results have been found in MR methods except IVW. The fourth issue pertains to the fact that the current GWAS research population is European populations, and this study has not studied other populations. Therefore, whether the research results can be extended to other people remains to be discussed. Moreover, Because Mendelian randomization studies necessitate IVs to be independent of confounding factors, certain IVs were omitted from the analysis. This exclusion has the potential to introduce bias and is acknowledged as a limitation of the study. Although all the F-statistics in this study are greater than 10, it is important to note that each variant has very low R2 values. This limitation should be emphasized. Finally, due to the limited types of inflammatory cytokines contained in the selected GWAS study, only 41 inflammatory cytokines were analyzed in this MR analysis, but not all inflammatory cytokines were analyzed.

## 5 Conclusion

Our study successfully established a causal association between genetically proxied circulating levels of IL-18, IL-1β, and RANTES and the risk of MDD. This significant finding not only contributes to a deeper comprehension of the pathogenesis of MDD but also holds promise for the development of effective clinical management strategies. Consequently, IL-18, IL-1β, and RANTES emerge as potential therapeutic targets for the prevention and treatment of MDD.

## Data Availability

The original contributions presented in the study are included in the article/Supplementary Material, further inquiries can be directed to the corresponding authors.
