# Supplementary material for: Genetically predicted circulating levels of cytokines and the risk of depression: a bidirectional Mendelian-randomization study
Source: Front Genet. 2023 Aug 4;14:1242614. doi: 10.3389/fgene.2023.1242614 (PMC10436531; doi:10.3389/fgene.2023.1242614)
Supplement: Supplementary file 2 [file Image1.pdf]

Figure 1 Scatter plot, funnel plot, and forest plot of SNPs associated with cytokines and their risk of depression.

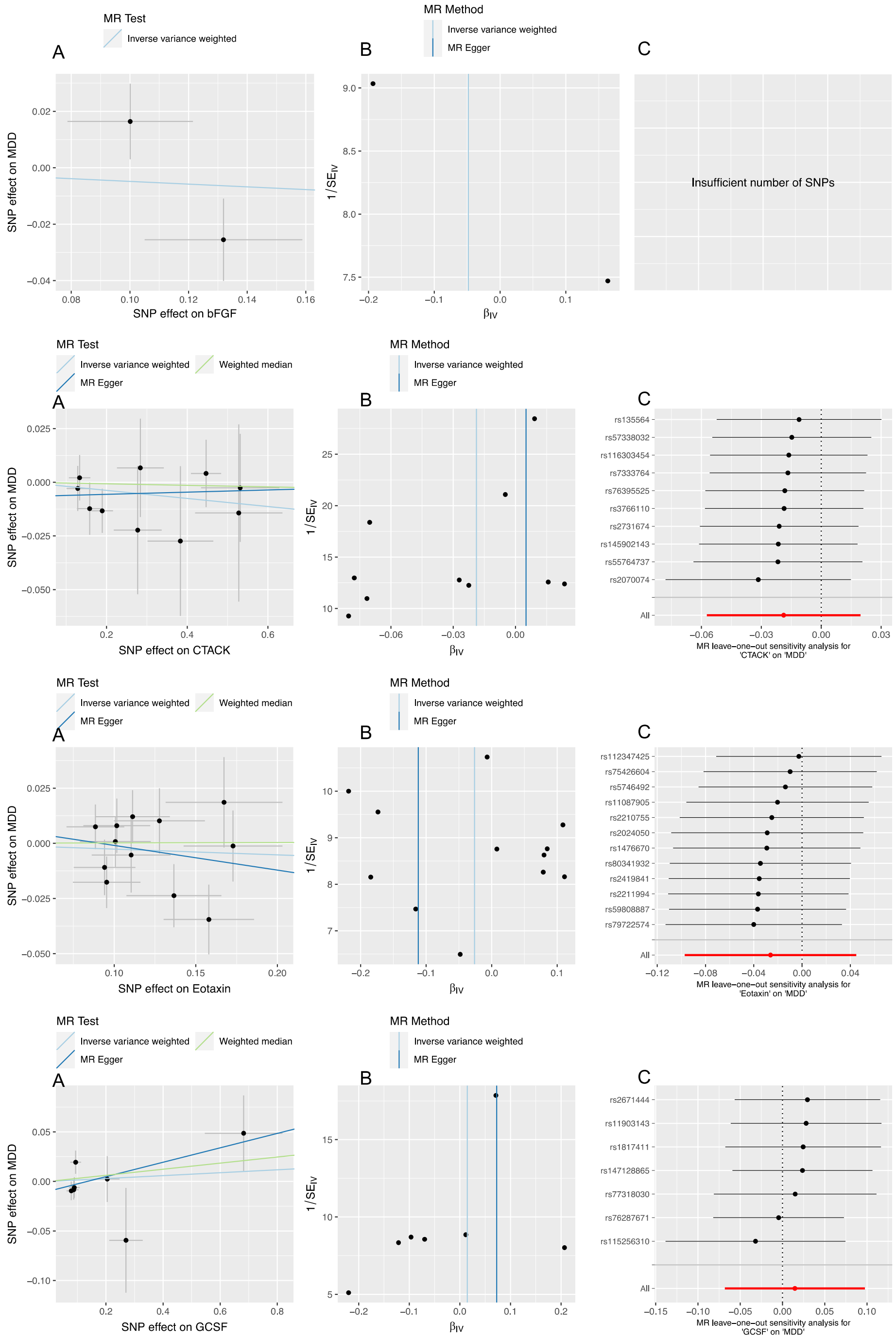

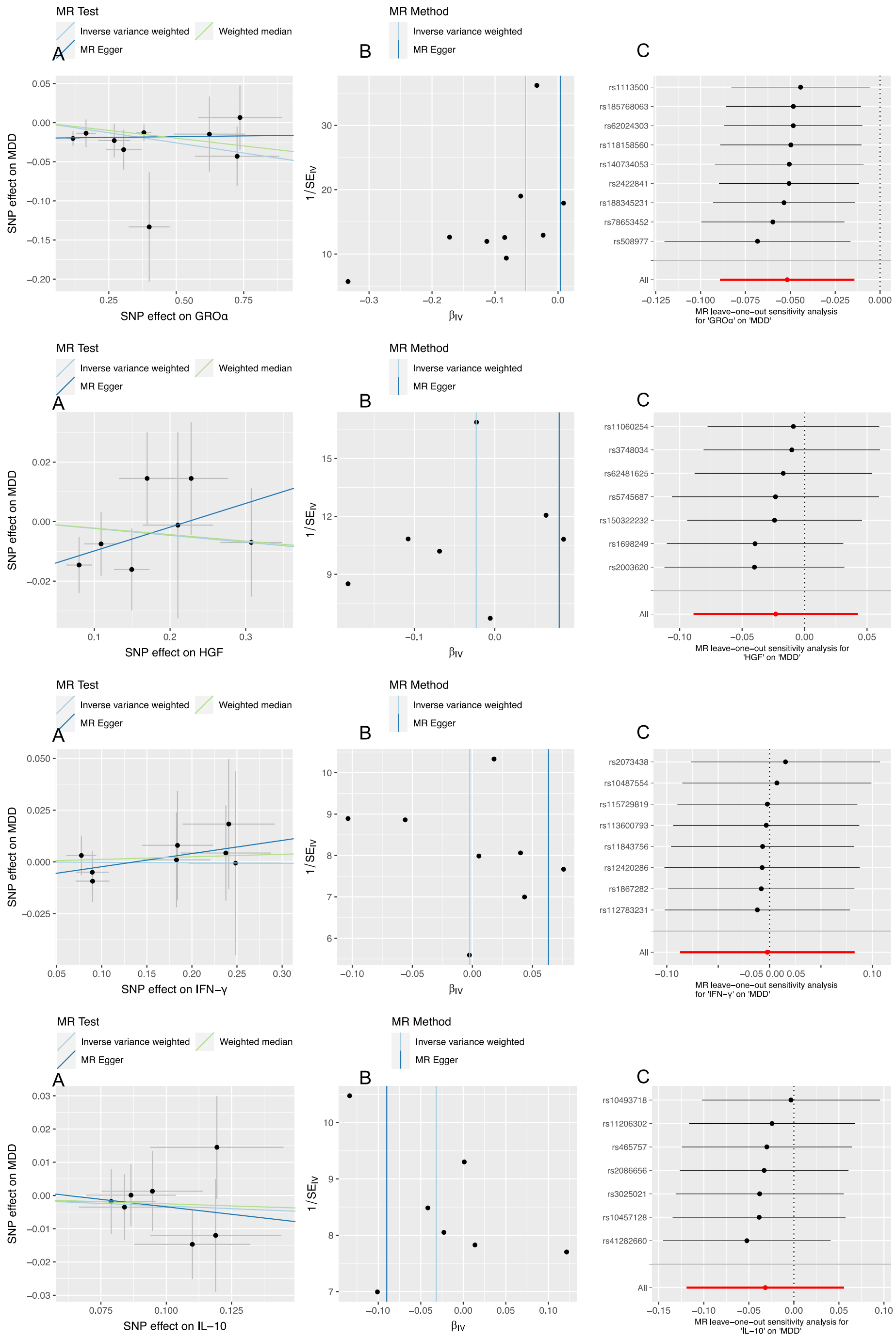
MR Test
Inverse variance weighted
Weighted median
MR Egger
MR Method
A
B
C
SNP effect on MDD
SNP effect on GROα
1/SE_IV
β_IV
rs1113500
rs185768063
rs62024303
rs118158560
rs140734053
rs2422841
rs188345231
rs78653452
rs508977
All
MR leave−one−out sensitivity analysis for 'GROα' on 'MDD'
SNP effect on HGF
rs11060254
rs3748034
rs62481625
rs5745687
rs150322232
rs1698249
rs2003620
MR leave−one−out sensitivity analysis for 'HGF' on 'MDD'
SNP effect on IFN−γ
rs2073438
rs10487554
rs115729819
rs113600793
rs11843756
rs12420286
rs1867282
rs112783231
MR leave−one−out sensitivity analysis for 'IFN−γ' on 'MDD'
SNP effect on IL−10
rs10493718
rs11206302
rs465757
rs2086656
rs3025021
rs10457128
rs41282660
MR leave−one−out sensitivity analysis for 'IL−10' on 'MDD'

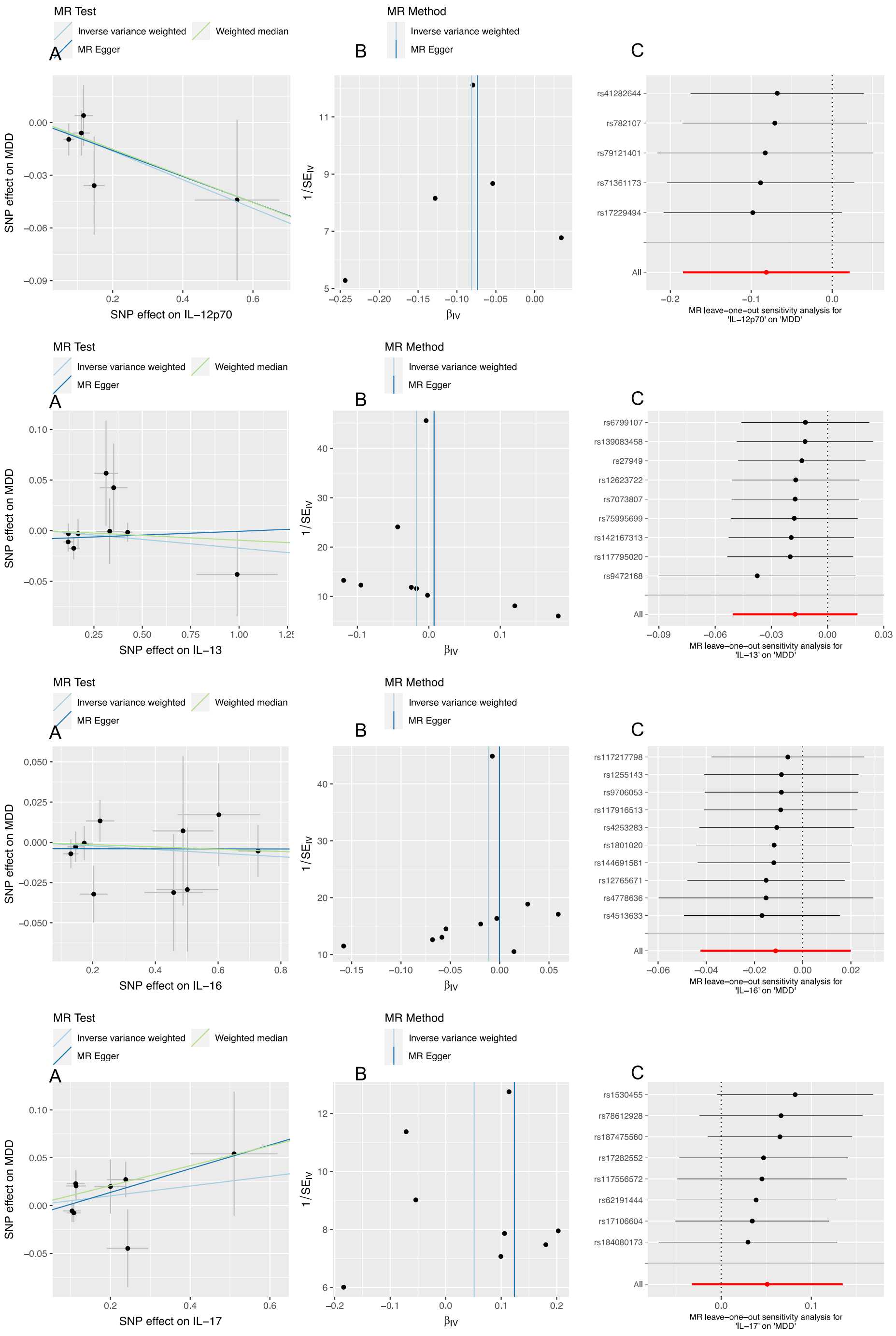
MR Test
Inverse variance weighted
Weighted median
MR Egger
MR Method
A
B
C
SNP effect on MDD
SNP effect on IL−12p70
1/SE_IV
β_IV
rs41282644
rs782107
rs79121401
rs71361173
rs17229494
All
MR leave−one−out sensitivity analysis for
'IL−12p70' on 'MDD'
SNP effect on IL−13
rs6799107
rs139083458
rs27949
rs12623722
rs7073807
rs75995699
rs142167313
rs117795020
rs9472168
MR leave−one−out sensitivity analysis for
'IL−13' on 'MDD'
SNP effect on IL−16
rs117217798
rs1255143
rs9706053
rs117916513
rs4253283
rs1801020
rs144691581
rs12765671
rs4778636
rs4513633
MR leave−one−out sensitivity analysis for
'IL−16' on 'MDD'
SNP effect on IL−17
rs1530455
rs78612928
rs187475560
rs17282552
rs117556572
rs62191444
rs17106604
rs184080173
MR leave−one−out sensitivity analysis for
'IL−17' on 'MDD'

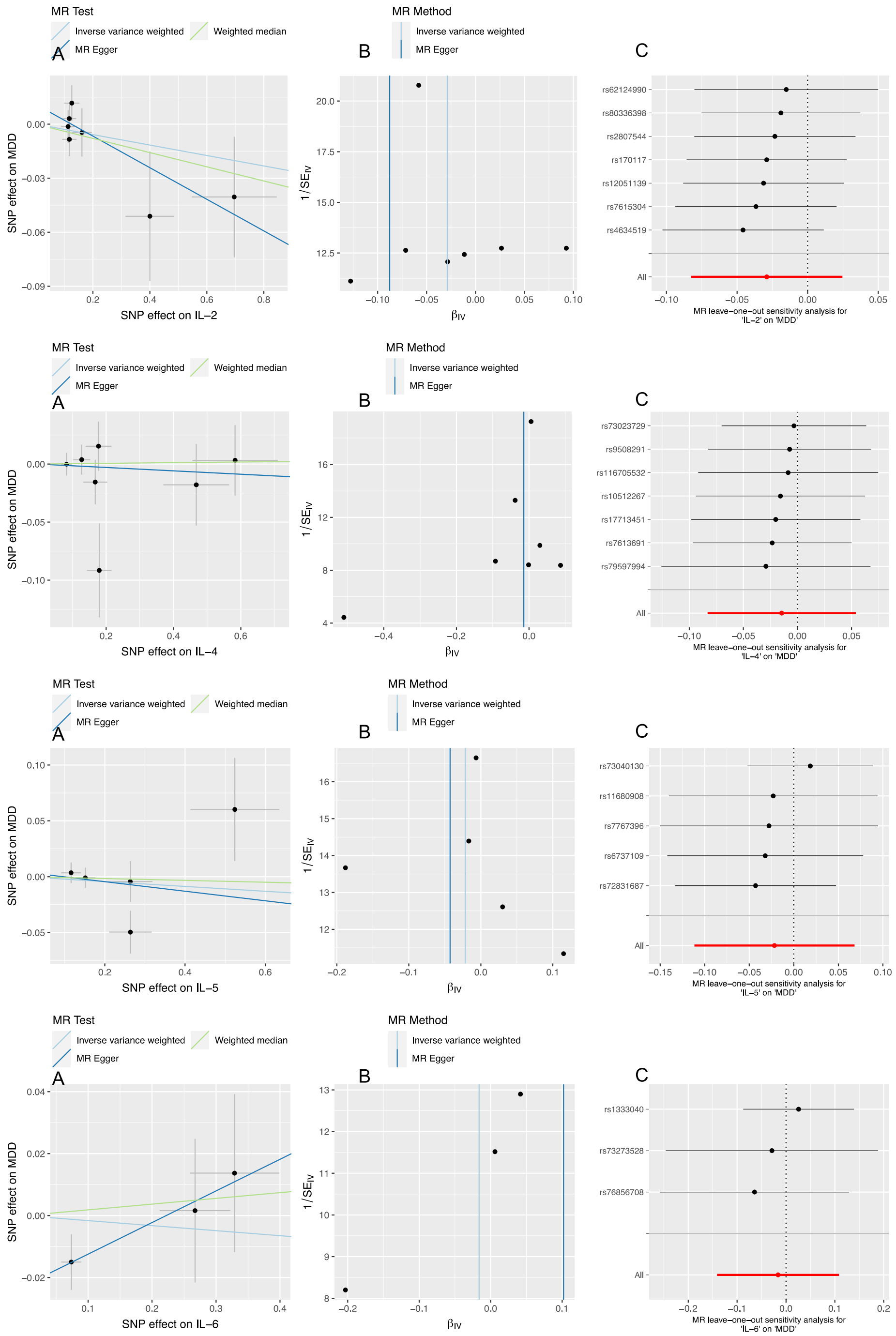
MR Test
Inverse variance weighted
Weighted median
MR Egger
MR Method
A
B
C
SNP effect on MDD
SNP effect on IL−2
1/SE$_{IV}$
β$_{IV}$
rs62124990
rs80336398
rs2807544
rs170117
rs12051139
rs7615304
rs4634519
All
MR leave−one−out sensitivity analysis for 'IL−2' on 'MDD'
SNP effect on IL−4
rs73023729
rs9508291
rs116705532
rs10512267
rs17713451
rs7613691
rs79597994
MR leave−one−out sensitivity analysis for 'IL−4' on 'MDD'
SNP effect on IL−5
rs73040130
rs11680908
rs7767396
rs6737109
rs72831687
MR leave−one−out sensitivity analysis for 'IL−5' on 'MDD'
SNP effect on IL−6
rs1333040
rs73273528
rs76856708
MR leave−one−out sensitivity analysis for 'IL−6' on 'MDD'

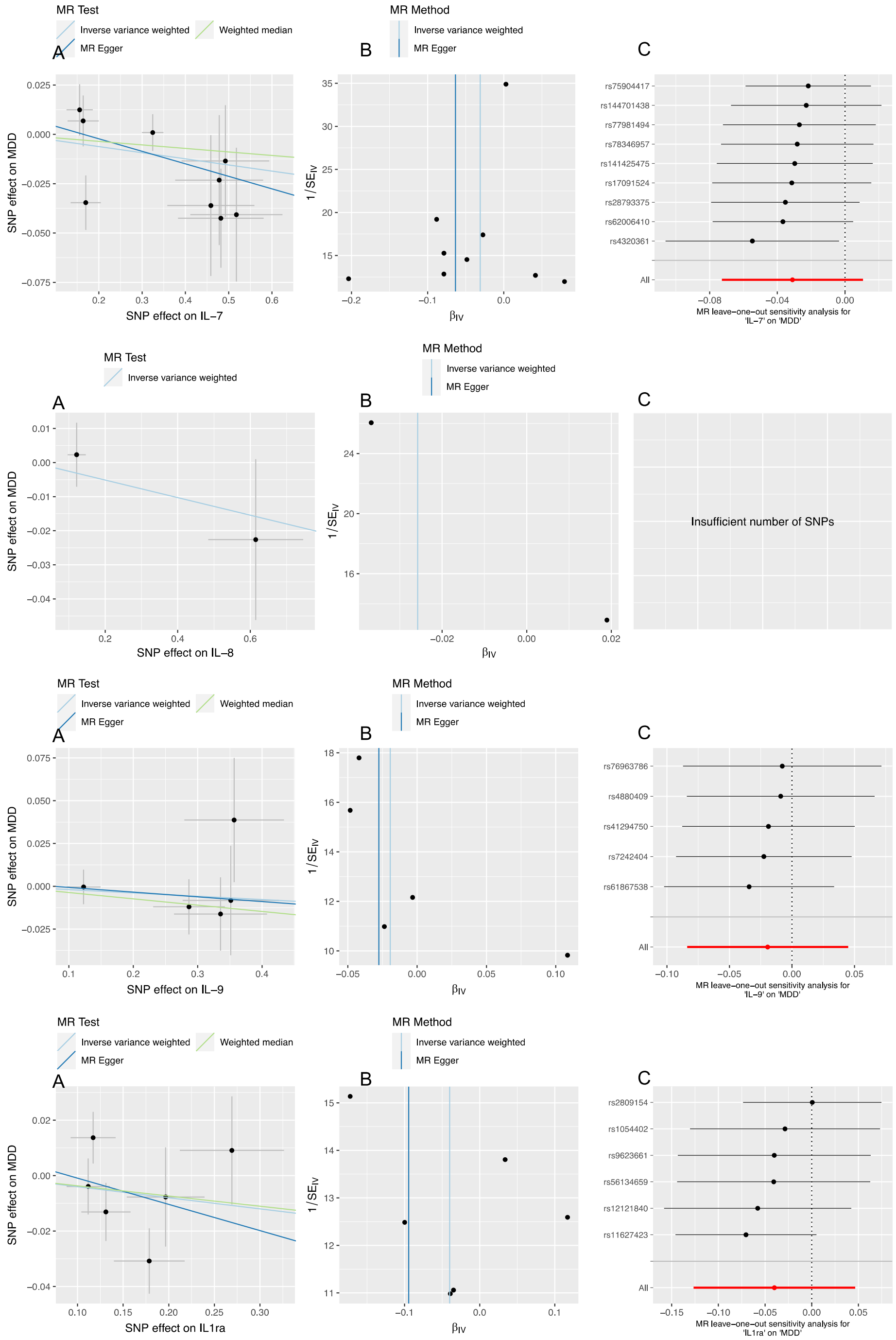
MR Test
Inverse variance weighted
Weighted median
MR Egger
MR Method
A
B
C
SNP effect on MDD
SNP effect on IL−7
1/SE$_{IV}$
β$_{IV}$
rs75904417
rs144701438
rs77981494
rs78346957
rs141425475
rs17091524
rs28793375
rs62006410
rs4320361
All
MR leave−one−out sensitivity analysis for 'IL−7' on 'MDD'
SNP effect on IL−8
Insufficient number of SNPs
SNP effect on IL−9
rs76963786
rs4880409
rs41294750
rs7242404
rs61867538
MR leave−one−out sensitivity analysis for 'IL−9' on 'MDD'
SNP effect on IL1ra
rs2809154
rs1054402
rs9623661
rs56134659
rs12121840
rs11627423
MR leave−one−out sensitivity analysis for 'IL1ra' on 'MDD'

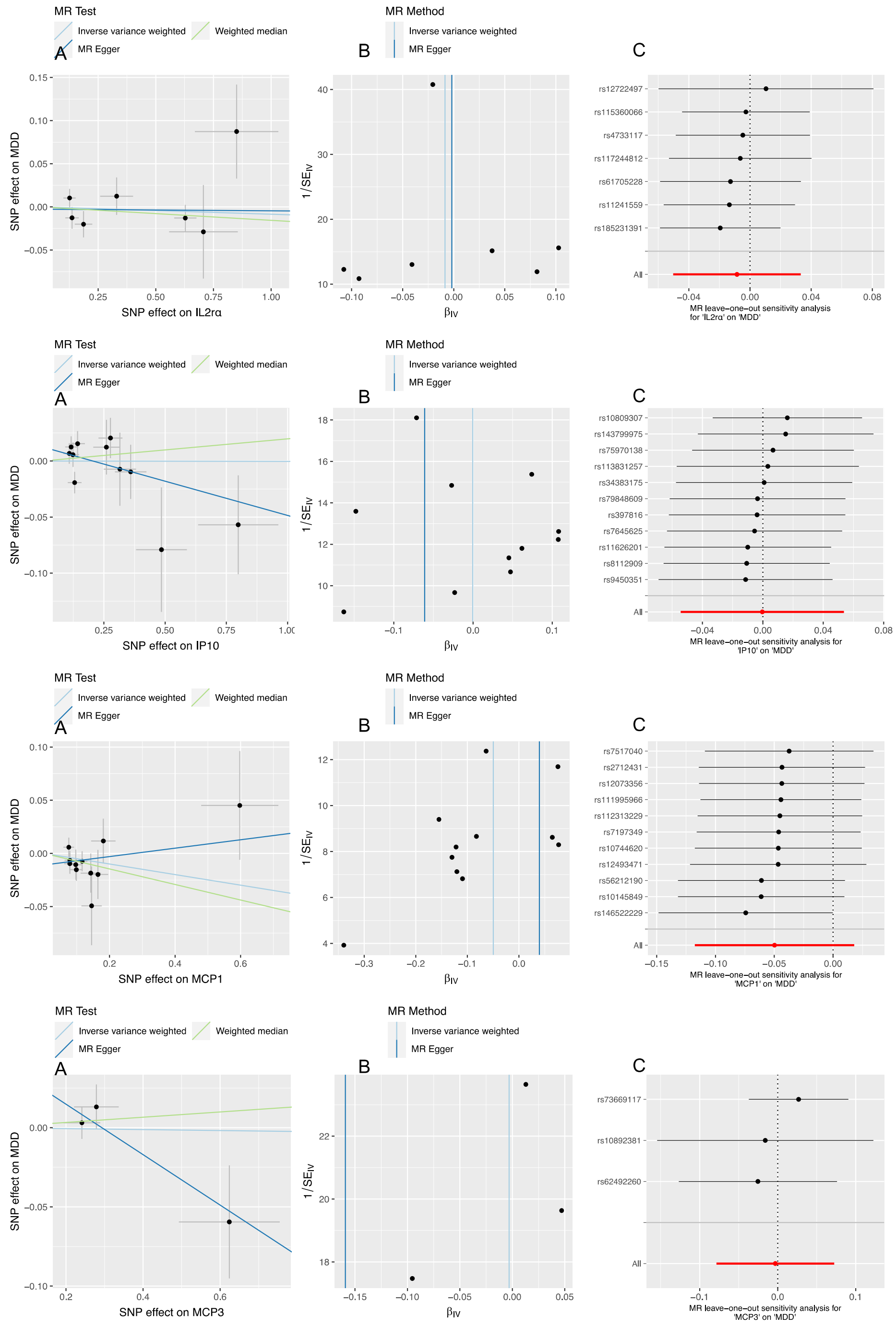
MR Test
Inverse variance weighted
Weighted median
MR Egger
MR Method
Inverse variance weighted
MR Egger
A
B
C
SNP effect on MDD
SNP effect on IL2rα
1/SE$_{IV}$
β_{IV}
rs12722497
rs115360066
rs4733117
rs117244812
rs61705228
rs11241559
rs185231391
All
MR leave−one−out sensitivity analysis for 'IL2rα' on 'MDD'
SNP effect on IP10
rs10809307
rs143799975
rs75970138
rs113831257
rs34383175
rs79848609
rs397816
rs7645625
rs11626201
rs8112909
rs9450351
All
MR leave−one−out sensitivity analysis for 'IP10' on 'MDD'
SNP effect on MCP1
rs7517040
rs2712431
rs12073356
rs111995966
rs112313229
rs7197349
rs10744620
rs12493471
rs56212190
rs10145849
rs146522229
All
MR leave−one−out sensitivity analysis for 'MCP1' on 'MDD'
SNP effect on MCP3
rs73669117
rs10892381
rs62492260
All
MR leave−one−out sensitivity analysis for 'MCP3' on 'MDD'

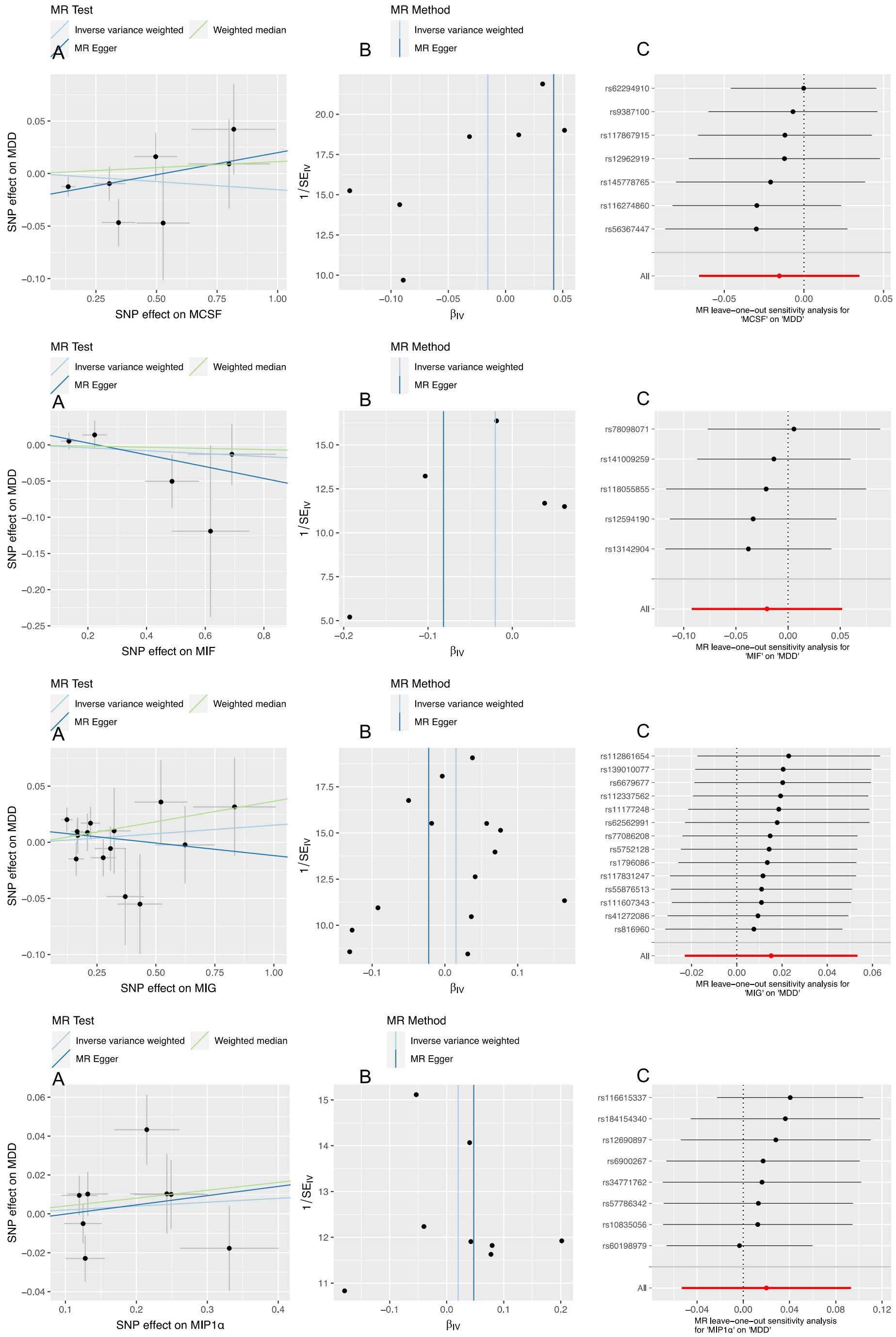

MR Test
Inverse variance weighted
Weighted median
MR Egger
A
SNP effect on MDD
SNP effect on MCSF
MR Method
B
1/SE_IV
β_IV
C
rs62294910
rs9387100
rs117867915
rs12962919
rs145778765
rs116274860
rs56367447
All
MR leave−one−out sensitivity analysis for 'MCSF' on 'MDD'
SNP effect on MIF
rs78098071
rs141009259
rs118055855
rs12594190
rs13142904
MR leave−one−out sensitivity analysis for 'MIF' on 'MDD'
SNP effect on MIG
rs112861654
rs139010077
rs6679677
rs112337562
rs11177248
rs62562991
rs77086208
rs5752128
rs1796086
rs117831247
rs55876513
rs111607343
rs41272086
rs816960
MR leave−one−out sensitivity analysis for 'MIG' on 'MDD'
SNP effect on MIP1α
rs116615337
rs184154340
rs12690897
rs6900267
rs34771762
rs57786342
rs10835056
rs60198979
MR leave−one−out sensitivity analysis for 'MIP1α' on 'MDD'

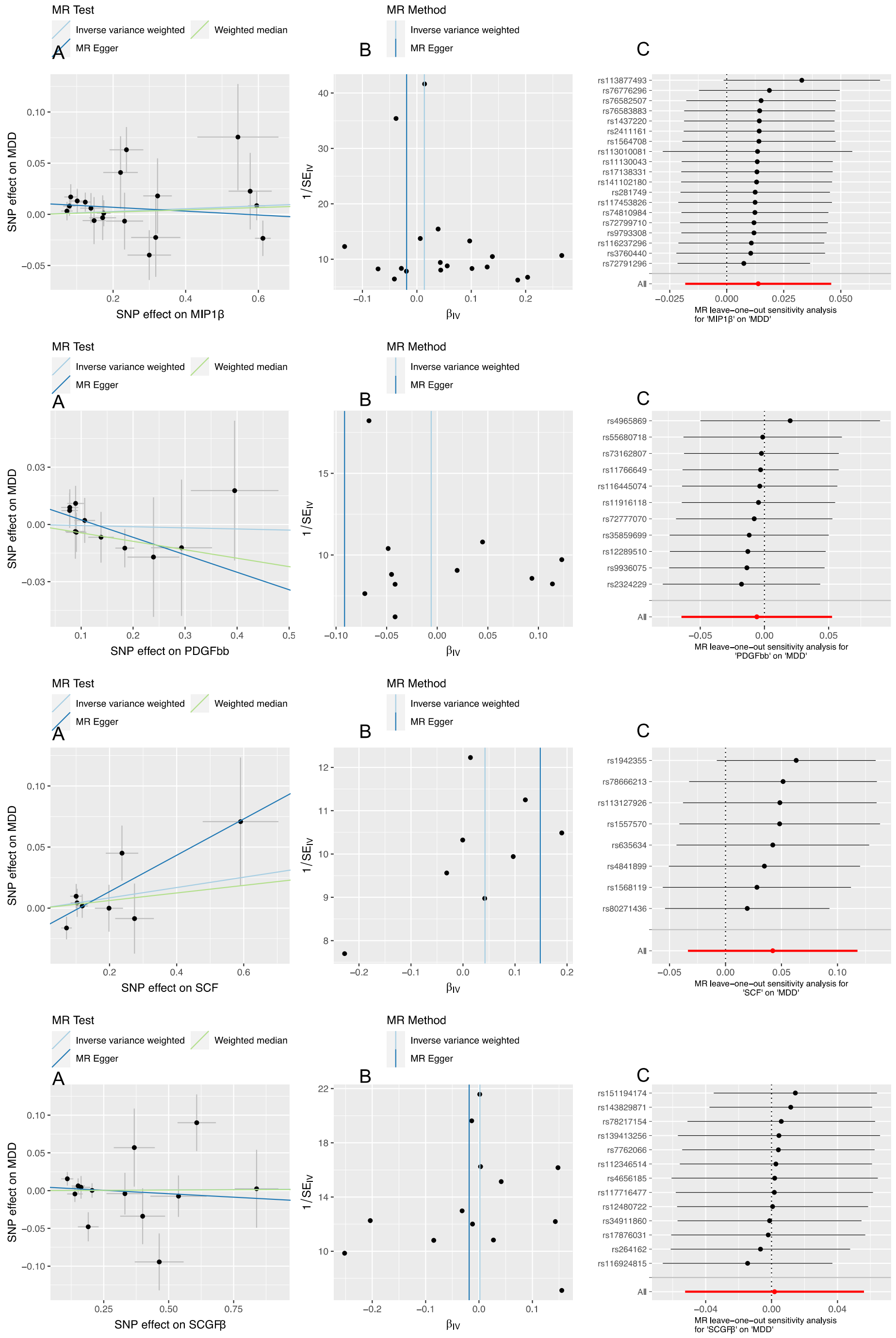

MR Test
Inverse variance weighted
Weighted median
MR Egger
MR Method
Inverse variance weighted
MR Egger
A
B
C
SNP effect on MDD
SNP effect on MIP1β
1/SE$_{IV}$
β$_{IV}$
rs113877493
rs76776296
rs76582507
rs76583883
rs1437220
rs2411161
rs1564708
rs113010081
rs11130043
rs17138331
rs141102180
rs281749
rs117453826
rs74810984
rs72799710
rs9793308
rs116237296
rs3760440
rs72791296
All
MR leave−one−out sensitivity analysis for 'MIP1β' on 'MDD'
SNP effect on PDGFbb
rs4965869
rs55680718
rs73162807
rs11766649
rs116445074
rs11916118
rs72777070
rs35859699
rs12289510
rs9936075
rs2324229
MR leave−one−out sensitivity analysis for 'PDGFbb' on 'MDD'
SNP effect on SCF
rs1942355
rs78666213
rs113127926
rs1557570
rs635634
rs4841899
rs1568119
rs80271436
MR leave−one−out sensitivity analysis for 'SCF' on 'MDD'
SNP effect on SCGFβ
rs151194174
rs143829871
rs78217154
rs139413256
rs7762066
rs112346514
rs4656185
rs117716477
rs12480722
rs34911860
rs17876031
rs264162
rs116924815
MR leave−one−out sensitivity analysis for 'SCGFβ' on 'MDD'

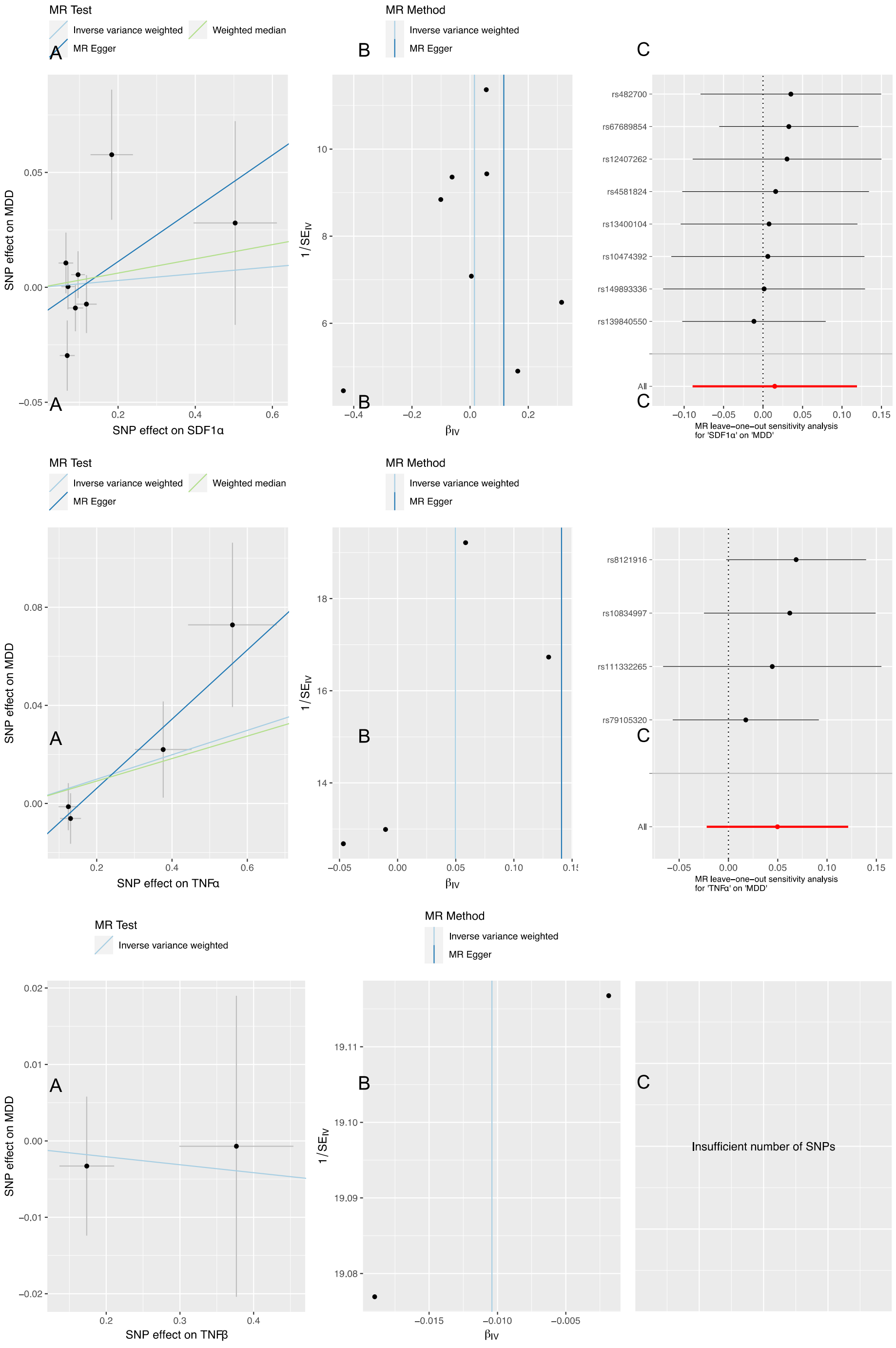
MR Test
Inverse variance weighted
Weighted median
MR Egger
A
SNP effect on MDD
SNP effect on SDF1α
MR Method
Inverse variance weighted
MR Egger
B
1/SE_IV
β_IV
C
rs482700
rs67689854
rs12407262
rs4581824
rs13400104
rs10474392
rs149893336
rs139840550
All
MR leave–one–out sensitivity analysis
for 'SDF1α' on 'MDD'
MR Test
Inverse variance weighted
Weighted median
MR Egger
A
SNP effect on MDD
SNP effect on TNFα
MR Method
Inverse variance weighted
MR Egger
B
1/SE_IV
β_IV
C
rs8121916
rs10834997
rs111332265
rs79105320
All
MR leave–one–out sensitivity analysis
for 'TNFα' on 'MDD'
MR Test
Inverse variance weighted
A
SNP effect on MDD
SNP effect on TNFβ
MR Method
Inverse variance weighted
MR Egger
B
1/SE_IV
β_IV
C
Insufficient number of SNPs

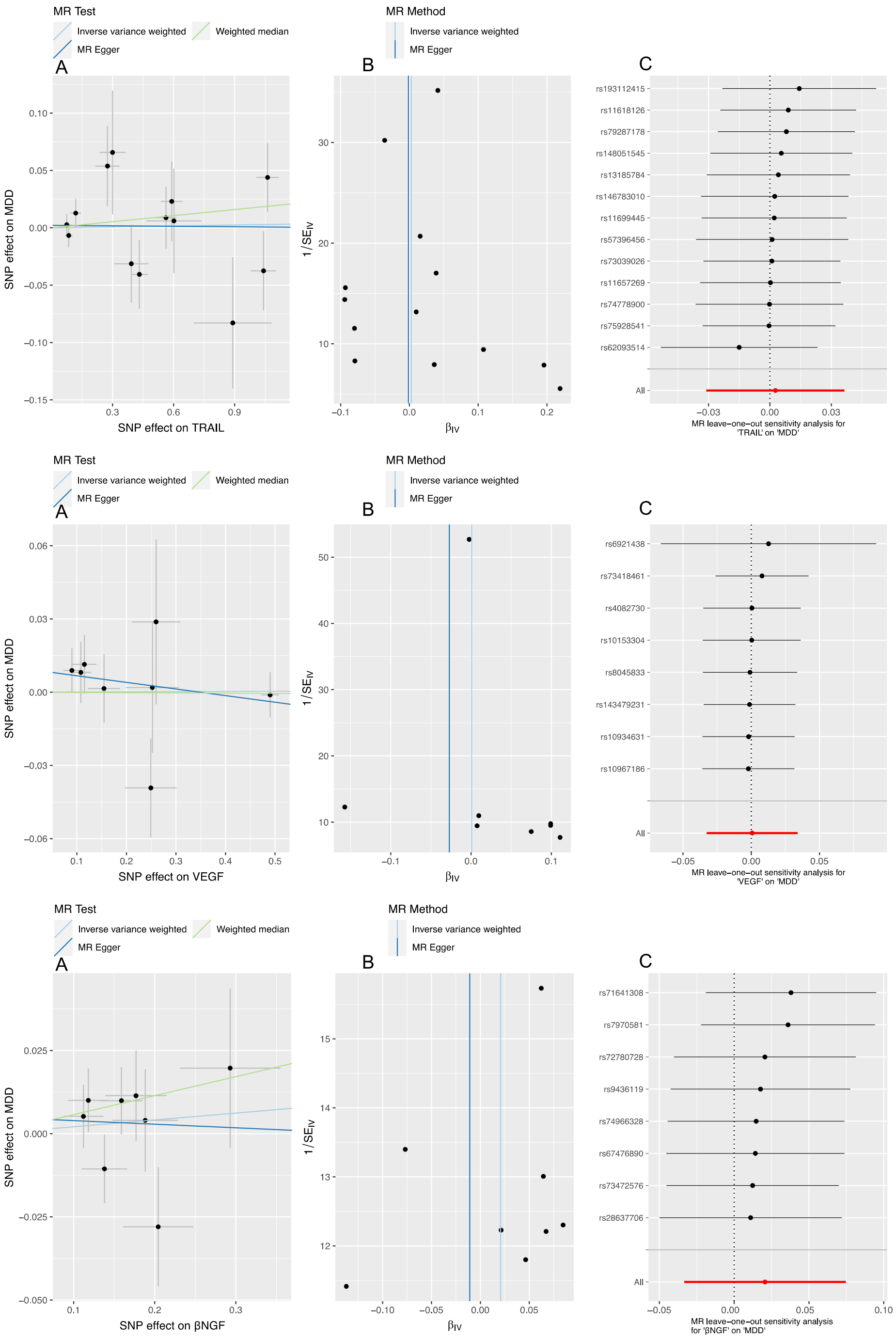

MR Test
Inverse variance weighted
Weighted median
MR Egger
A
SNP effect on MDD
SNP effect on TRAIL
MR Method
Inverse variance weighted
MR Egger
B
1/SE$_{IV}$
β_{IV}
C
rs193112415
rs11618126
rs79287178
rs148051545
rs13185784
rs146783010
rs11699445
rs57396456
rs73039026
rs11657269
rs74778900
rs75928541
rs62093514
All
MR leave−one−out sensitivity analysis for 'TRAIL' on 'MDD'
MR Test
Inverse variance weighted
Weighted median
MR Egger
A
SNP effect on MDD
SNP effect on VEGF
MR Method
Inverse variance weighted
MR Egger
B
1/SE$_{IV}$
β_{IV}
C
rs6921438
rs73418461
rs4082730
rs10153304
rs8045833
rs143479231
rs10934631
rs10967186
All
MR leave−one−out sensitivity analysis for 'VEGF' on 'MDD'
MR Test
Inverse variance weighted
Weighted median
MR Egger
A
SNP effect on MDD
SNP effect on βNGF
MR Method
Inverse variance weighted
MR Egger
B
1/SE$_{IV}$
β_{IV}
C
rs71641308
rs7970581
rs72780728
rs9436119
rs74966328
rs67476890
rs73472576
rs28637706
All
MR leave−one−out sensitivity analysis for 'βNGF' on 'MDD'

Figure2 Scatter plot, funnel plot, and forest plot of SNPs associated with depression and their risk of cytokines.

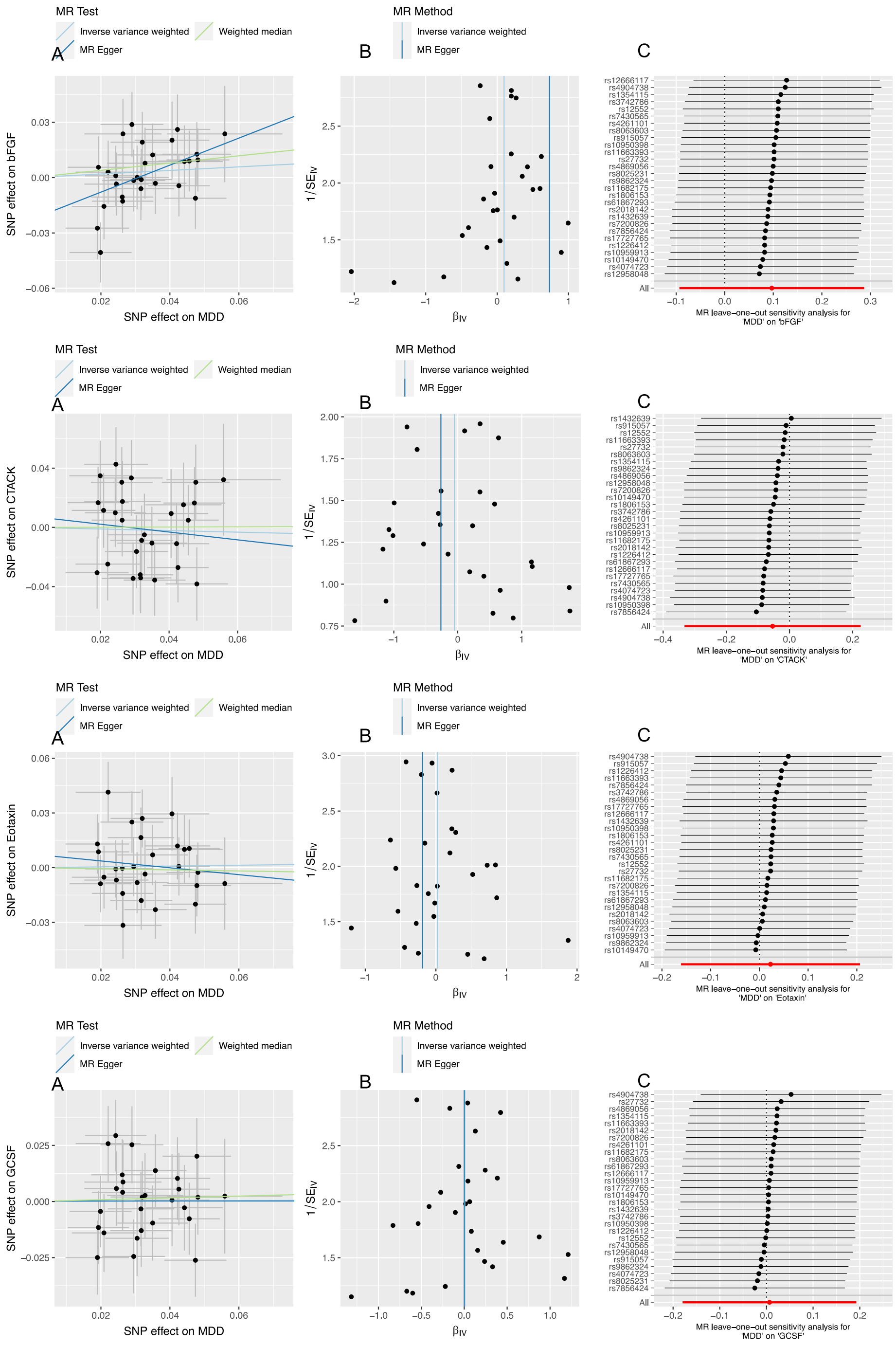

MR Test
Inverse variance weighted
Weighted median
MR Egger
MR Method
A
B
C
SNP effect on bFGF
SNP effect on MDD
1/SE_IV
β_IV
MR leave−one−out sensitivity analysis for 'MDD' on 'bFGF'
SNP effect on CTACK
MR leave−one−out sensitivity analysis for 'MDD' on 'CTACK'
SNP effect on Eotaxin
MR leave−one−out sensitivity analysis for 'MDD' on 'Eotaxin'
SNP effect on GCSF
MR leave−one−out sensitivity analysis for 'MDD' on 'GCSF'
All

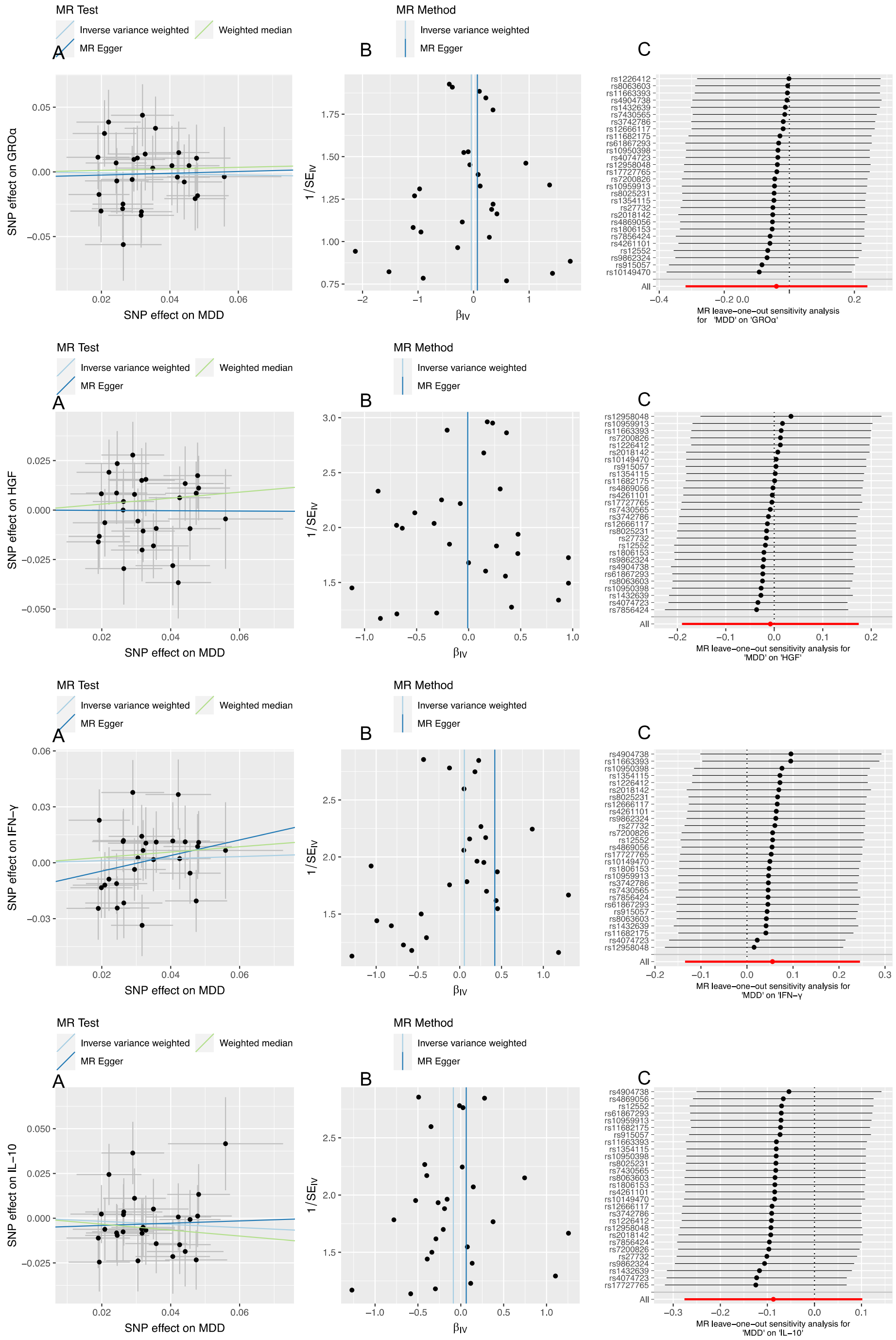
MR Test
Inverse variance weighted
Weighted median
MR Egger
MR Method
Inverse variance weighted
MR Egger
A
B
C
SNP effect on GROα
SNP effect on MDD
1/SE_IV
β_IV
MR leave-one-out sensitivity analysis for 'MDD' on 'GROα'
SNP effect on HGF
MR leave-one-out sensitivity analysis for 'MDD' on 'HGF'
SNP effect on IFN-γ
MR leave-one-out sensitivity analysis for 'MDD' on 'IFN-γ
SNP effect on IL-10
MR leave-one-out sensitivity analysis for 'MDD' on 'IL-10'
All

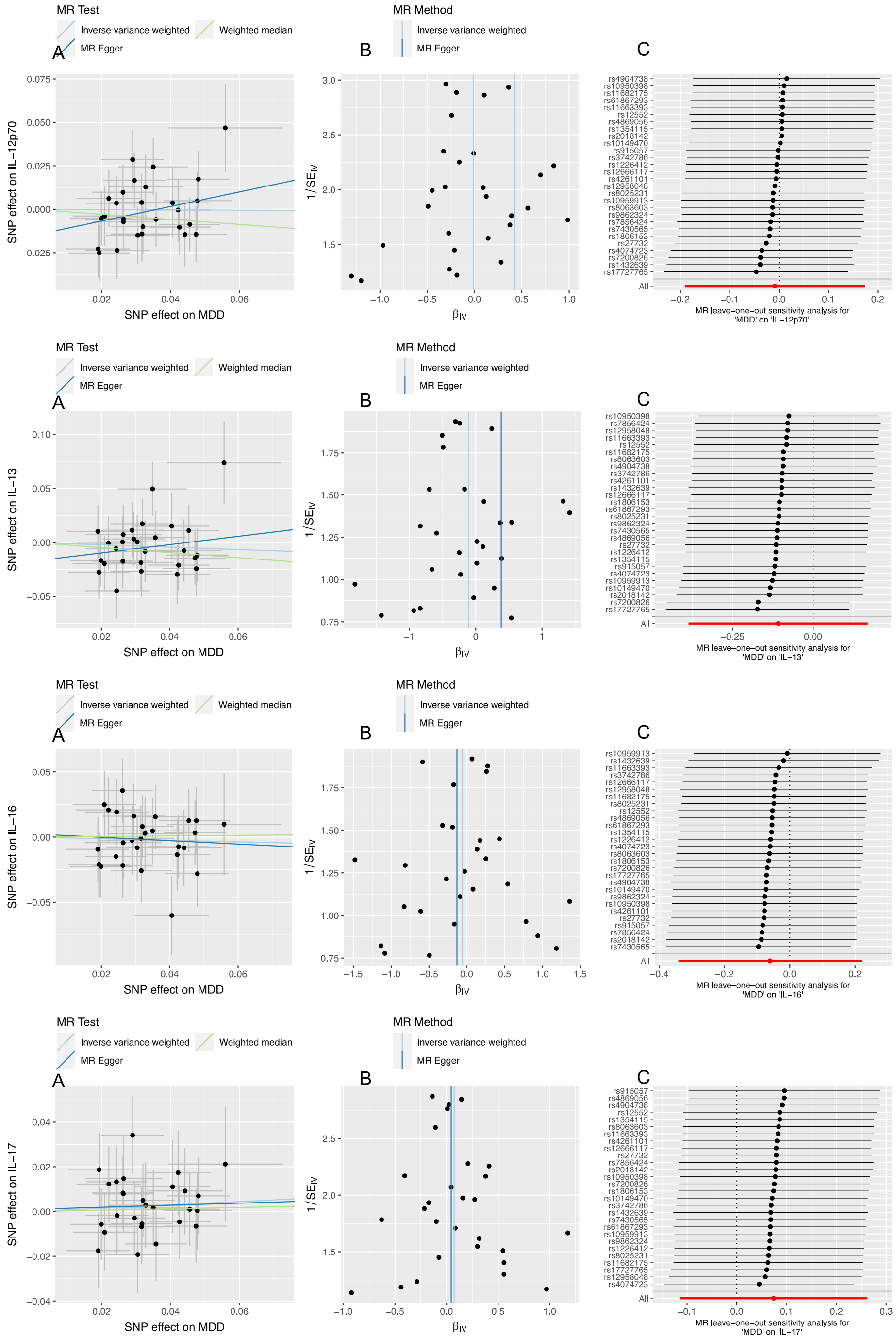

MR Test
Inverse variance weighted
Weighted median
MR Egger
MR Method
A
B
C
SNP effect on IL−12p70
SNP effect on MDD
1/SE$_{IV}$
β_{IV}
MR leave−one−out sensitivity analysis for 'MDD' on 'IL−12p70'
SNP effect on IL−13
MR leave−one−out sensitivity analysis for 'MDD' on 'IL−13'
SNP effect on IL−16
MR leave−one−out sensitivity analysis for 'MDD' on 'IL−16'
SNP effect on IL−17
MR leave−one−out sensitivity analysis for 'MDD' on 'IL−17'
All

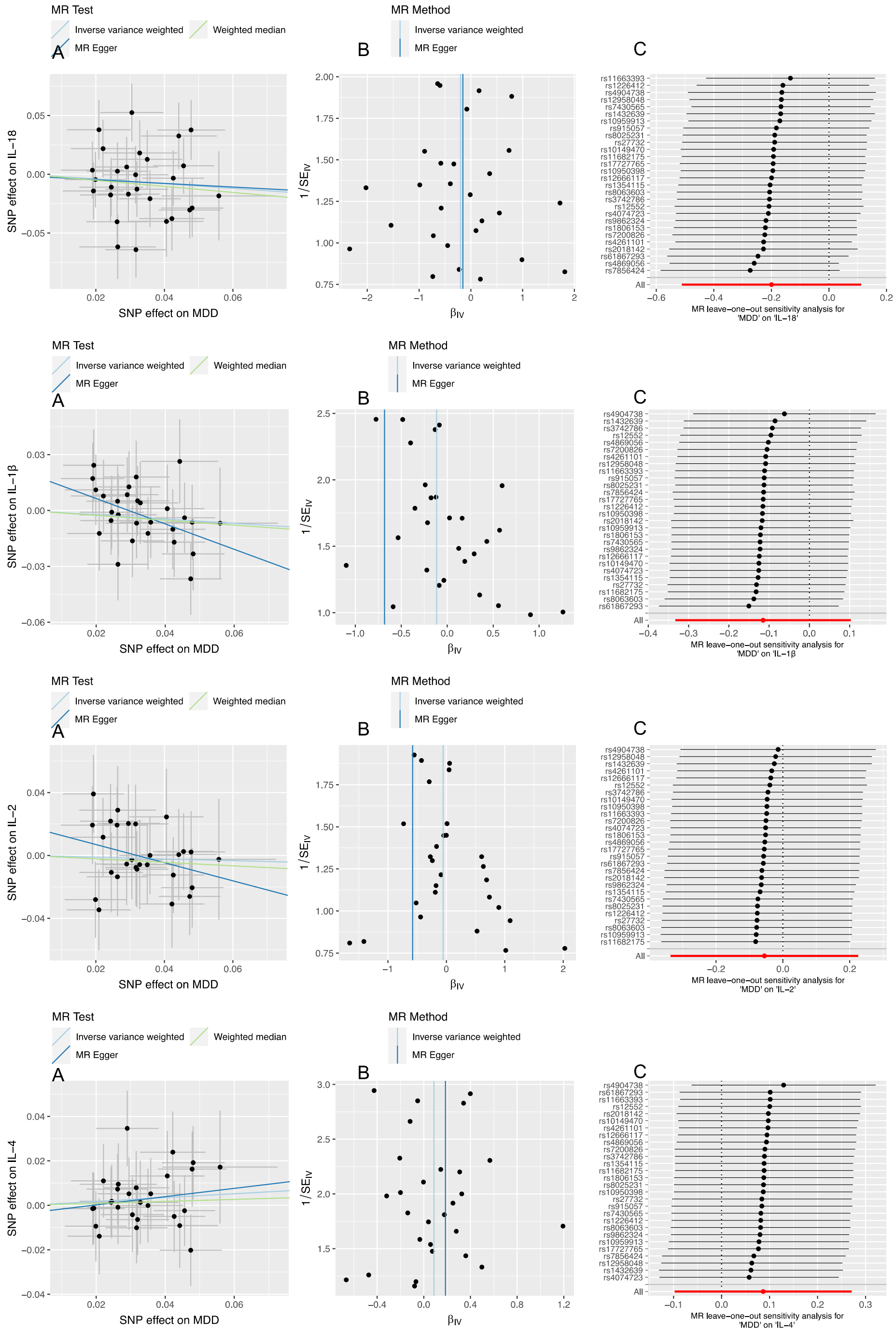
MR Test
Inverse variance weighted
Weighted median
MR Egger
MR Method
A
B
C
SNP effect on IL−18
SNP effect on MDD
1/SE_IV
β_IV
MR leave−one−out sensitivity analysis for
'MDD' on 'IL−18'
SNP effect on IL−1β
MR leave−one−out sensitivity analysis for
'MDD' on 'IL−1β'
SNP effect on IL−2
MR leave−one−out sensitivity analysis for
'MDD' on 'IL−2'
SNP effect on IL−4
MR leave−one−out sensitivity analysis for
'MDD' on 'IL−4'
All

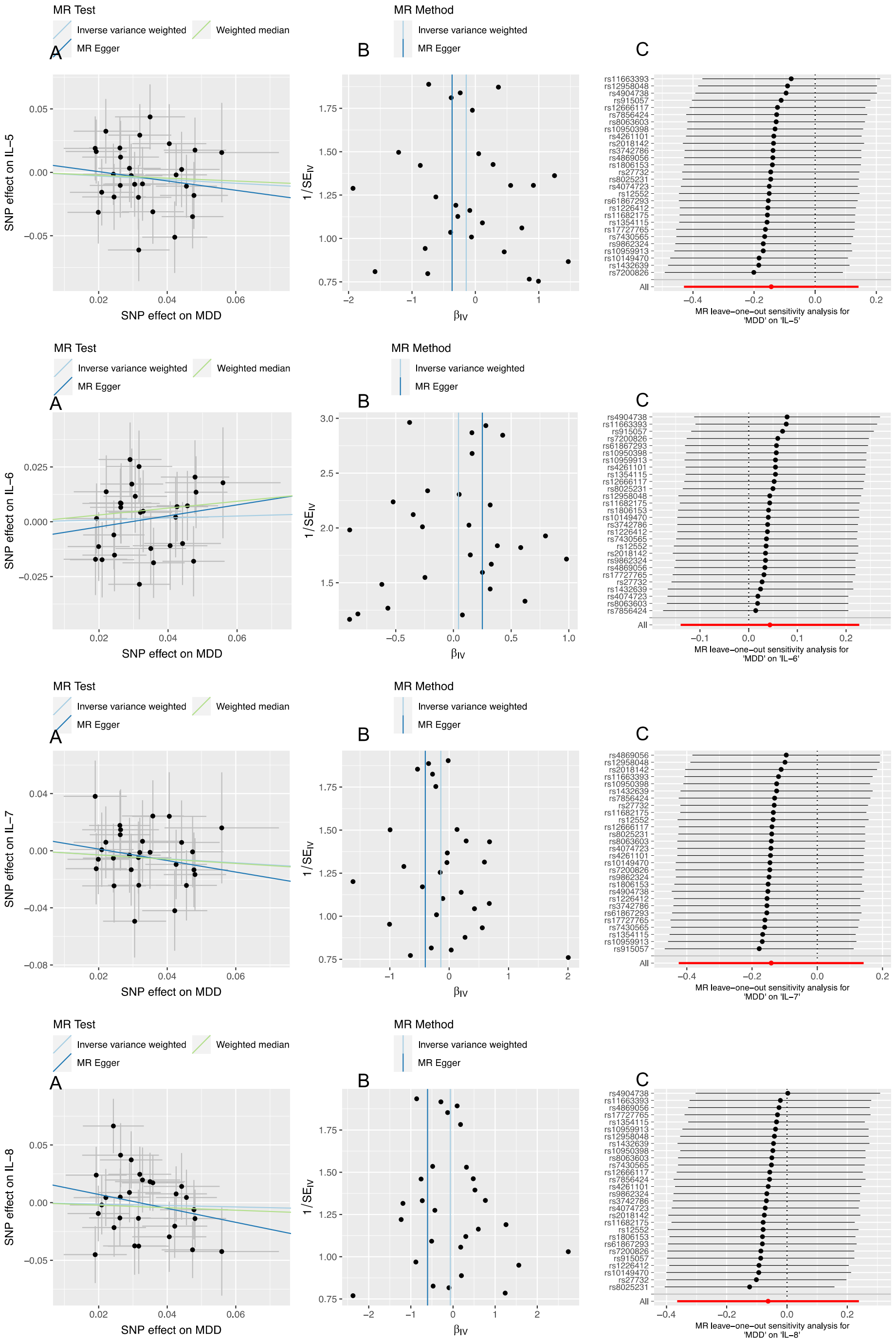
MR Test
Inverse variance weighted
Weighted median
MR Egger
MR Method
A
B
C
SNP effect on IL−5
SNP effect on IL−6
SNP effect on IL−7
SNP effect on IL−8
SNP effect on MDD
1/SE_IV
β_IV
MR leave−one−out sensitivity analysis for 'MDD' on 'IL−5'
MR leave−one−out sensitivity analysis for 'MDD' on 'IL−6'
MR leave−one−out sensitivity analysis for 'MDD' on 'IL−7'
MR leave−one−out sensitivity analysis for 'MDD' on 'IL−8'
All

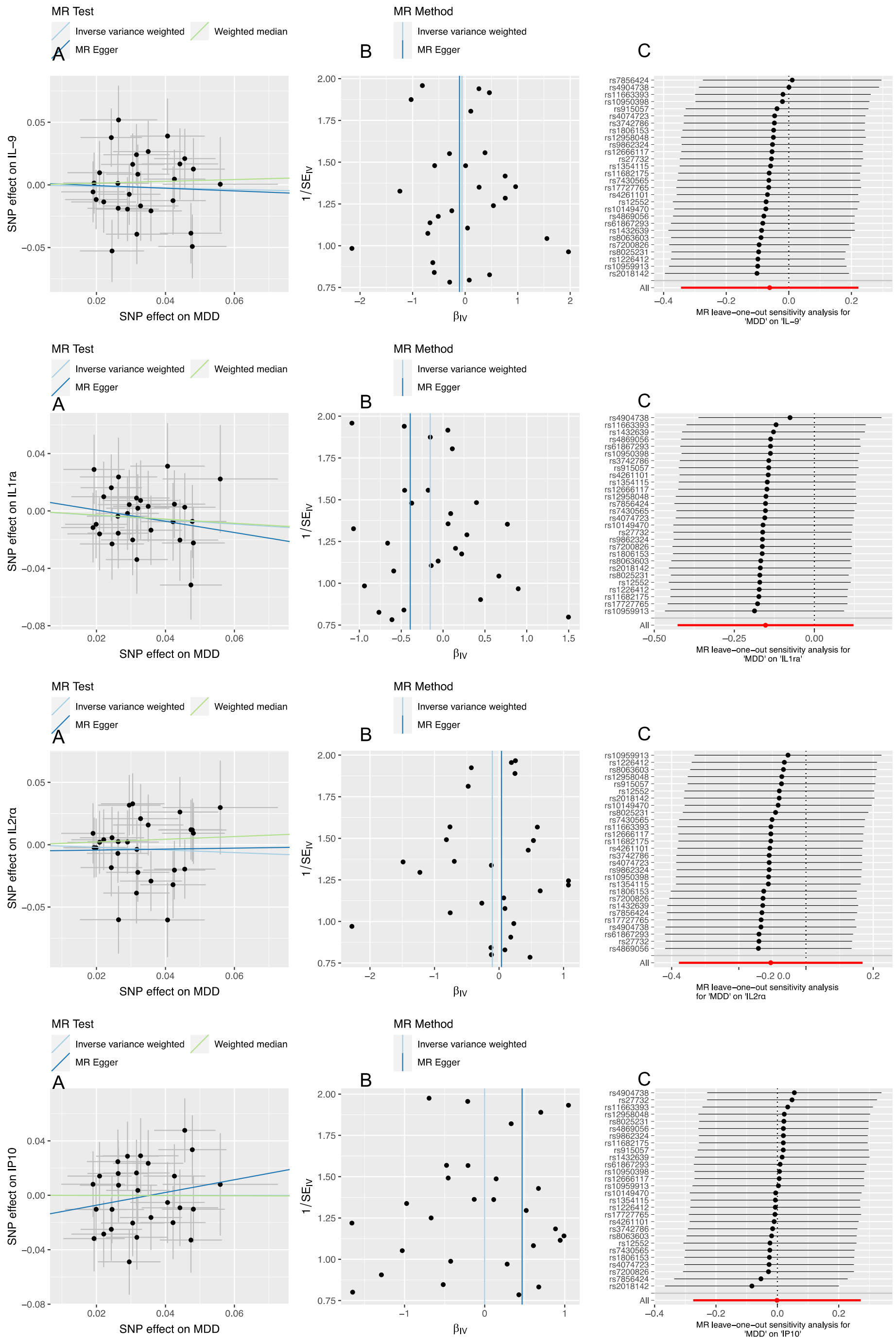

MR Test
Inverse variance weighted
Weighted median
MR Egger
MR Method
Inverse variance weighted
MR Egger
A
B
C
SNP effect on IL−9
SNP effect on MDD
1/SE_IV
β_IV
MR leave−one−out sensitivity analysis for 'MDD' on 'IL−9'
SNP effect on IL1ra
MR leave−one−out sensitivity analysis for 'MDD' on 'IL1ra'
SNP effect on IL2rα
MR leave−one−out sensitivity analysis for 'MDD' on 'IL2rα
SNP effect on IP10
MR leave−one−out sensitivity analysis for 'MDD' on 'IP10'
All

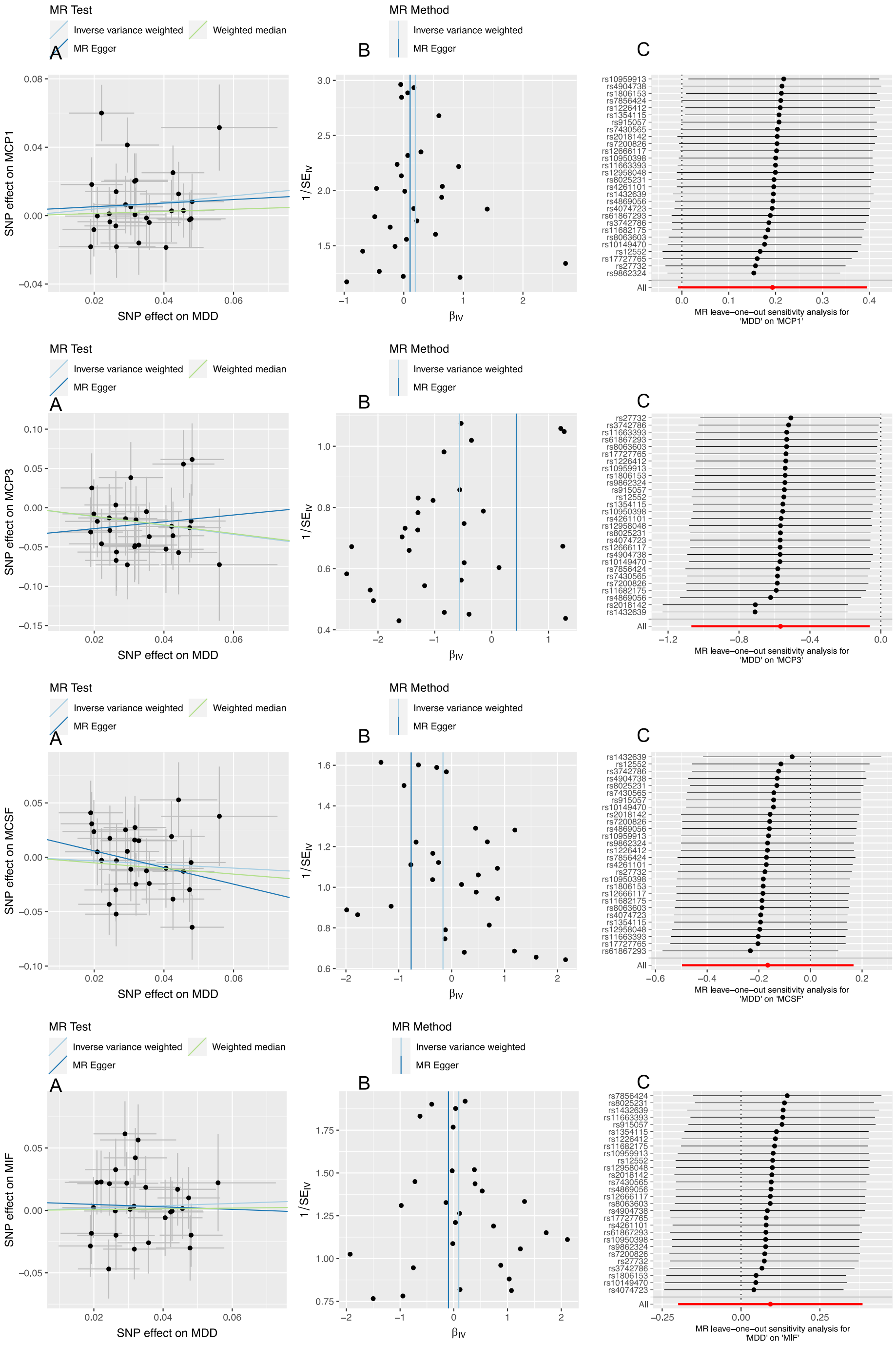

MR Test
Inverse variance weighted
Weighted median
MR Egger
MR Method
Inverse variance weighted
MR Egger
A
B
C
SNP effect on MCP1
SNP effect on MDD
1/SE_IV
β_IV
MR leave−one−out sensitivity analysis for
'MDD' on 'MCP1'
SNP effect on MCP3
MR leave−one−out sensitivity analysis for
'MDD' on 'MCP3'
SNP effect on MCSF
MR leave−one−out sensitivity analysis for
'MDD' on 'MCSF'
SNP effect on MIF
MR leave−one−out sensitivity analysis for
'MDD' on 'MIF'
All

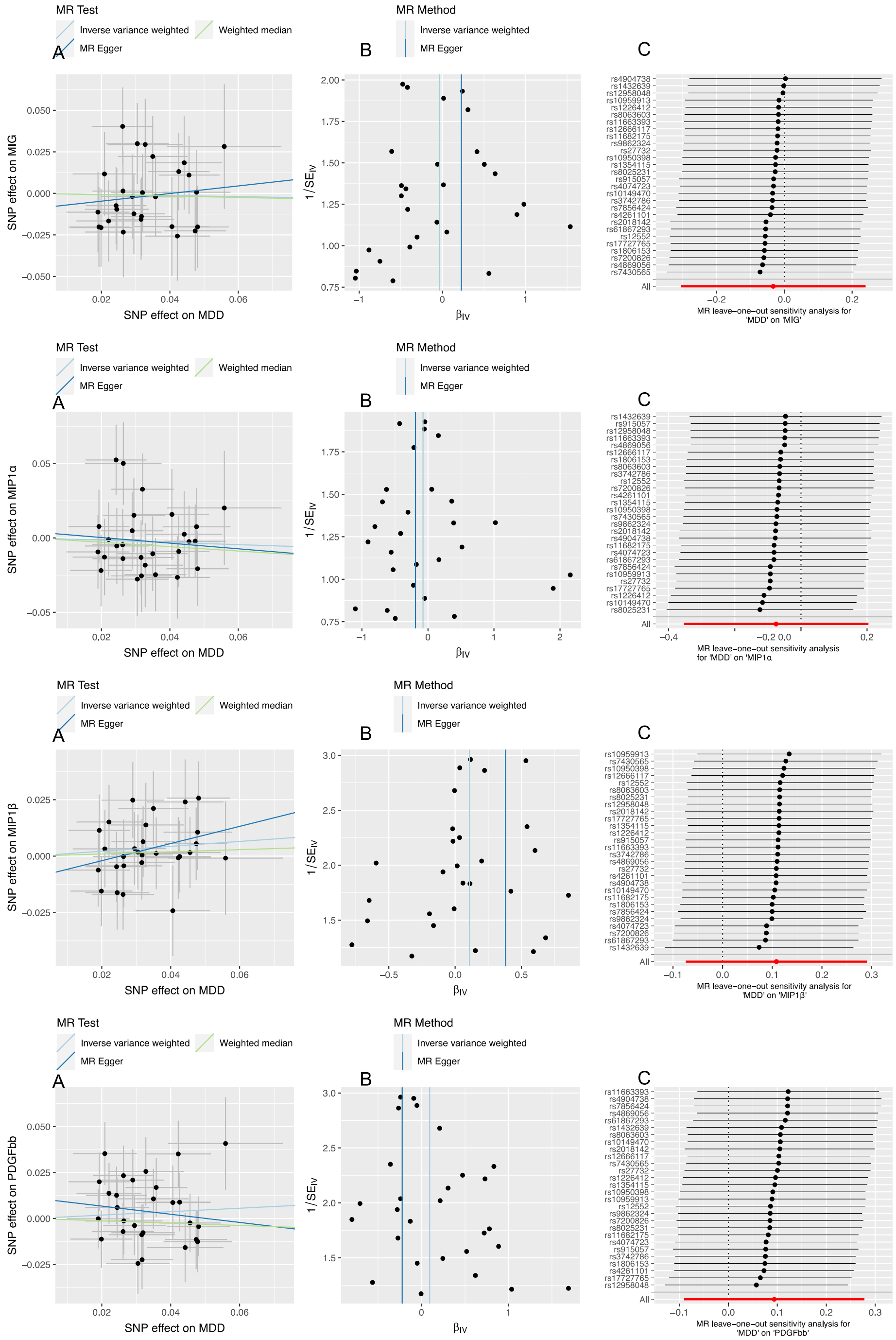

MR Test
Inverse variance weighted
Weighted median
MR Egger
MR Method
Inverse variance weighted
MR Egger
A
B
C
SNP effect on MIG
SNP effect on MDD
1/SE_IV
β_IV
MR leave−one−out sensitivity analysis for 'MDD' on 'MIG'
SNP effect on MIP1α
MR leave−one−out sensitivity analysis for 'MDD' on 'MIP1α'
SNP effect on MIP1β
MR leave−one−out sensitivity analysis for 'MDD' on 'MIP1β'
SNP effect on PDGFbb
MR leave−one−out sensitivity analysis for 'MDD' on 'PDGFbb'
All

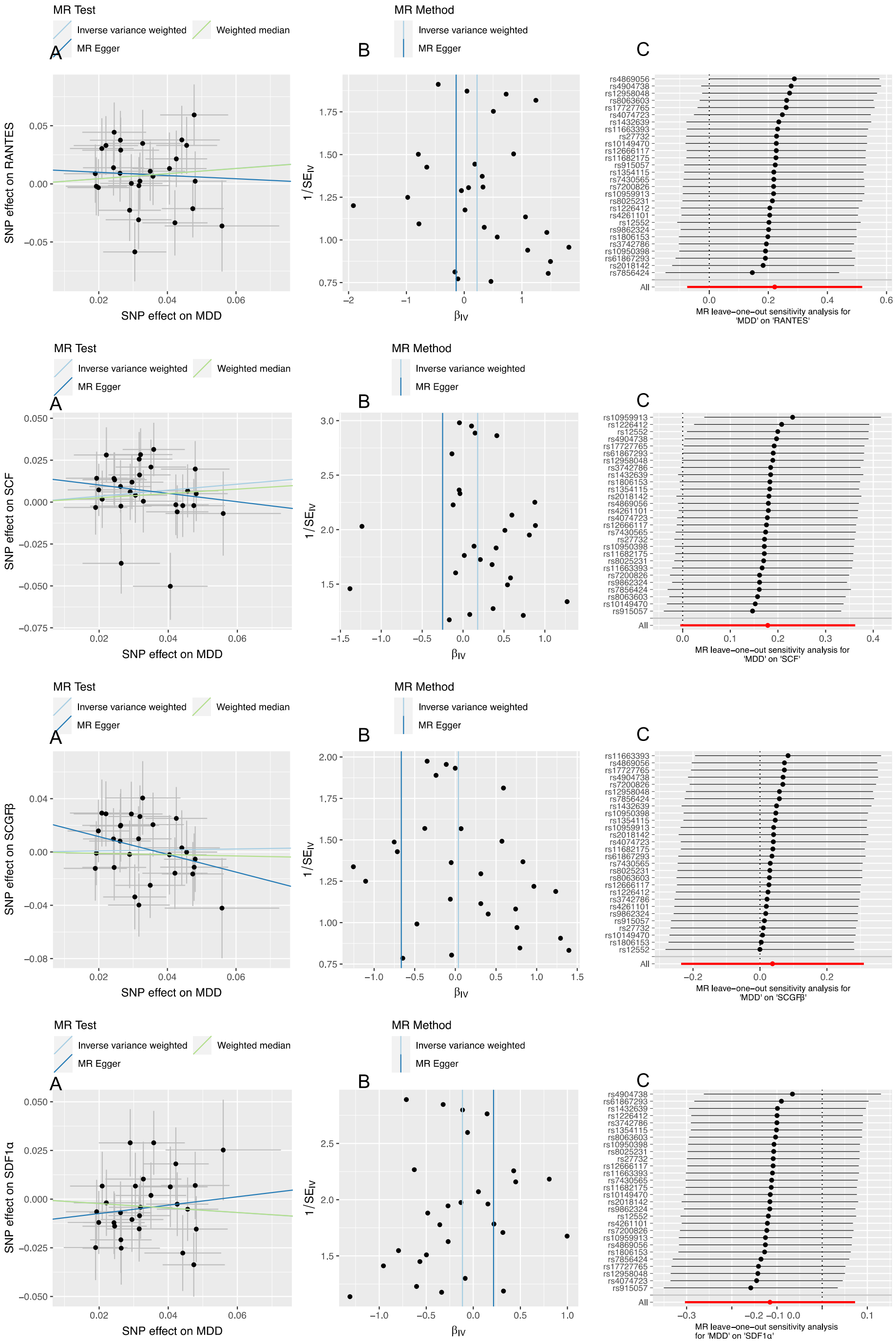
MR Test
Inverse variance weighted
Weighted median
MR Egger
A
SNP effect on RANTES
SNP effect on MDD
MR Method
Inverse variance weighted
MR Egger
B
1/SE_IV
β_IV
C
MR leave–one–out sensitivity analysis for 'MDD' on 'RANTES'
MR Test
Inverse variance weighted
Weighted median
MR Egger
A
SNP effect on SCF
SNP effect on MDD
MR Method
Inverse variance weighted
MR Egger
B
1/SE_IV
β_IV
C
MR leave–one–out sensitivity analysis for 'MDD' on 'SCF'
MR Test
Inverse variance weighted
Weighted median
MR Egger
A
SNP effect on SCGFβ
SNP effect on MDD
MR Method
Inverse variance weighted
MR Egger
B
1/SE_IV
β_IV
C
MR leave–one–out sensitivity analysis for 'MDD' on 'SCGFβ'
MR Test
Inverse variance weighted
Weighted median
MR Egger
A
SNP effect on SDF1α
SNP effect on MDD
MR Method
Inverse variance weighted
MR Egger
B
1/SE_IV
β_IV
C
MR leave–one–out sensitivity analysis for 'MDD' on 'SDF1α'
All

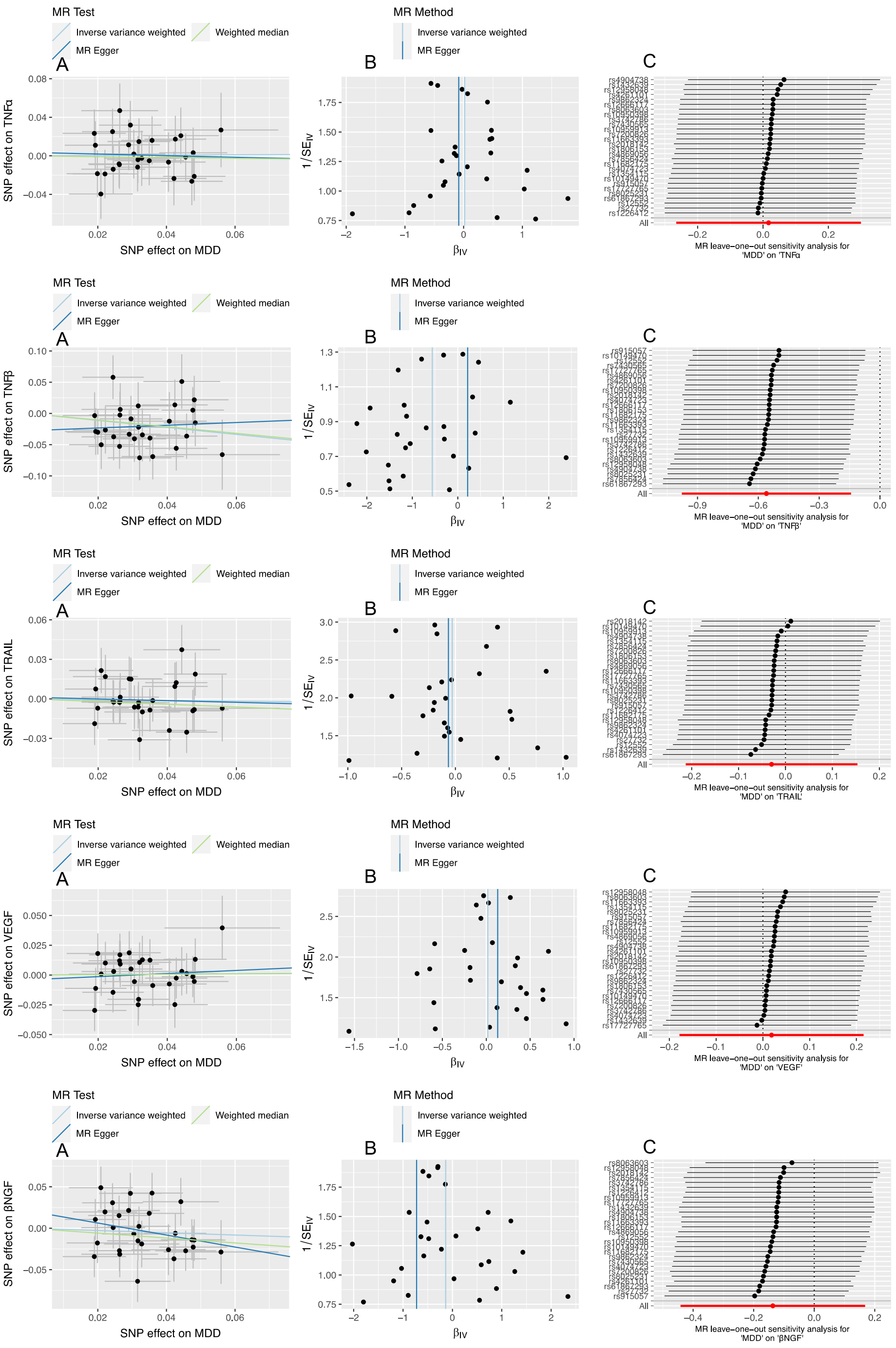

MR Test
Inverse variance weighted
Weighted median
MR Egger
MR Method
A
B
C
SNP effect on TNFα
SNP effect on TNFβ
SNP effect on TRAIL
SNP effect on VEGF
SNP effect on βNGF
SNP effect on MDD
1/SE_IV
β_IV
All
MR leave−one−out sensitivity analysis for 'MDD' on 'TNFα'
MR leave−one−out sensitivity analysis for 'MDD' on 'TNFβ'
MR leave−one−out sensitivity analysis for 'MDD' on 'TRAIL'
MR leave−one−out sensitivity analysis for 'MDD' on 'VEGF'
MR leave−one−out sensitivity analysis for 'MDD' on 'βNGF'
